# ACSF2-PGK1 interaction promotes ferroptosis in renal tubular epithelial cells of diabetic nephropathy by regulating Keap1/Nrf2 signaling

**DOI:** 10.1080/13510002.2025.2529618

**Published:** 2025-07-16

**Authors:** Xinran Liu, Chaoyi Chen, Sai Zhu, Xiaomei Luo, Li Gao, Ziyun Hu, Yu Ma, Ling Jiang, Yonggui Wu

**Affiliations:** a Department of Nephropathy, the First Affiliated Hospital of Anhui Medical University, Hefei, People’s Republic of China; b Center for Scientific Research, Anhui Medical University, Hefei, People’s Republic of China

**Keywords:** Diabetic nephropathy, ACSF2, renal tubular epithelial cell, ferroptosis, oxidative stress, mitochondrial‌, PGK1, Phosphorylation

## Abstract

**Objectives:**

Recent studies have highlighted the strong association between kidney disease and ferroptosis. However, the role of ferroptosis in diabetic nephropathy (DN) remains unclear. This study aimed to determine the role of ACSF2 in renal tubule injury in DN and its underlying mechanisms.

**Methods:**

We established diabetic kidney disease models both in vivo, using db/db mice, and in vitro, using high glucose induced HK-2 cells.

**Results:**

A significant upregulation of ACSF2 was observed in the renal tubules of patients with DN and db/db mice. ACSF2 expression correlated with renal tubule injury and renal function, indicating its potential as an independent biomarker in patients with DN. Silencing ACSF2 alleviated high glucose-induced renal tubular epithelial cell injury by reducing oxidative stress-induced ferroptosis in vivo and in vitro. Mechanistically, liquid chromatography-tandem mass spectrometry and co-immunoprecipitation demonstrated that ACSF2 specifically binds to PGK1. ACSF2 affected Keap1 dimerization by regulating PGK1 phosphorylation at serine 203, which subsequently affects the levels of NRF2. Moreover, PGK1 stabilizes ACSF2 via deubiquitination, establishing a positive feedback loop. The results provide evidence that the interaction between ACSF2 and PGK1 promotes DN progression by regulating oxidative stress-induced ferroptosis.

**Discussion:**

ACSF2 participates in crosstalk between oxidative stress and ferroptosis. This could be a potential therapeutic target for DN.

## Introduction

Diabetic nephropathy (DN) is the primary contributor to end-stage renal disease [1,2]. With the global increase in diabetes prevalence, the DN burden is also expected to increase, highlighting the need for effective prevention and management strategies [3,4]. The mechanisms underlying DN pathogenesis have not been fully elucidated and remain limited [5,6], highlighting the urgent need to discover new biomarkers and therapeutic targets for its diagnosis and treatment.

Ferroptosis is a non-apoptotic form of cell death that is regulated by reactive oxygen species (ROS), distinguished from apoptosis by its characteristic iron accumulation and lipid peroxidation (LPO) [7,8]. Recently, ferroptosis was identified as a key player in DN progression, particularly in tubular epithelial cells. Targeting ferroptosis is an innovative therapeutic strategy for treating DN [9, 10]. Acyl-CoA Synthetase family member 2 (ACSF2) contributes to the production of polyunsaturated fatty acids (PUFAs); moreover, PUFA peroxidation is a hallmark ferroptosis event [11, 12]. ACSF2 is crucial in maintaining cellular redox balance. It modulates ROS production by regulating lipid metabolic pathways and mitochondrial function [8,13]. Growing evidence suggests that ACSF2 regulates ferroptosis and is involved in several diseases [11,14,15]. However, its role in DN pathogenesis remains unclear.

In this study, ACSF2 was found to be upregulated in tubular cells using renal biopsy samples from patients with DN and renal tissues from db/db mice. Furthermore, we demonstrated its role in ferroptosis regulation in renal tubular cells in vivo and in vitro. Mechanistic studies demonstrated that ACSF2 promoted PGK1 phosphorylation at serine 203, whereas PGK1 deubiquitinated and stabilized ACSF2. The ACSF2-PGK1 interaction reduced cellular antioxidant ability and sensitized cells to ferroptosis by regulating the Keap1/Nrf2 axis. ACSF2 may serve as a biomarker for predicting DN progression and as a therapeutic target for inhibiting its progression.

## Materials and methods

### Reagents and materials

Antibodies included anti-ACSF2 (16140-1-AP, Proteintech), anti-APQ1 (sc-25287, Santa Cruz Biotechnology), anti-CD28K (14479-1-AP, Proteintech), anti-APQ3 (sc-518001, Santa Cruz Biotechnology), anti-CD31 (11265-1-AP, Proteintech), anti-synaptopodin (sc-515842, Santa Cruz Biotechnology), anti-PDGFR-β (sc-374573, Santa Cruz Biotechnology), anti-neutrophil gelatinase-associated lipocalin (NGAL, ab125075, Abcam), anti-kidney injury molecule-1 (KIM-1, ab78494, Abcam), anti-glutathione peroxidase 4 (GPX4, ab125066, Abcam), anti-4-hydroxynonenal (4-HNE, ab48506, Abcam), anti-phosphoglycerate kinase 1 (PGK1, 17811-1-AP, Proteintech), anti-Ubiquitin (20326, Cell Signaling Technology), anti-PGK1 pS203 (SAB487P, Signalway Antibody), anti-kelch-like ECH-associated protein 1 (Keap1, ab139729, Abcam), anti-nuclear factor erythroid 2-related factor 2 (Nrf2, 16396-1-AP, Proteintech) and anti-heme oxygenase 1 (HO-1,10701-1-AP, Proteintech). The periodic acid-Schiff (PAS), enzyme-linked immunosorbent assay, and iron content assay kits were purchased from Solarbio (G1281), Abcam Biotechnology (Cambridge, UK; ab207620), and LeaGene (Beijing, China), respectively. The glutathione (GSH), malondialdehyde (MDA), superoxide dismutase (SOD), and LPO assay kits were obtained from Nanjing Jiancheng Bioengineering Institute (Nanjing, China).

### Study population

Renal tissue samples were collected at the First Affiliated Hospital of Anhui Medical University. Patients with tumors, infections, or autoimmune diseases were excluded. Eighty-three patients with renal biopsy-proven DN and type 2 diabetes were enrolled, and 35 patients with biopsy-proven minor glomerular lesions (MCD) were selected as control participants. Twenty-one paracancerous renal tissues were also collected. A detailed description of all participants is provided in Table S1. This study was approved by the Medical Ethics Committee of the First Affiliated Hospital of Anhui Medical University (PJ2023-10-27).

### RNA sequencing analysis (RNA-seq)

Renal tissues from four patients diagnosed with DN by renal biopsy and four paracancerous renal tissues were collected for RNA-seq. RNA-seq was performed according to a previously established protocol [16].

### Animals

Six-week-old male BKS-db/db mice (*n* = 36) were purchased from Gempharmatech Co. Ltd. (Nanjing, China). For adeno-associated virus serotype 9 (AAV9)-mediated ACSF2 knockdown in db/db mice, 100 µL of AAV9-packaged ACSF2 knockdown plasmid with a 1 × 10^12^ vg/mL, containing the Ksp-cadherin promoter, was injected into the tail vein at 9 weeks of age. At 22 weeks of age, all mice were euthanized following anesthesia. The renal tissues were collected for pathological testing, western blotting, and immunohistochemistry. The animal study was approved by the Ethics Committee for Animal Research of the Anhui Medical University (LLSC20241775).

### Immunohistochemistry

Paraffin sections were dewaxed, dehydrated, and subjected to antigen retrieval with citrate buffer (P0081; Beyotime) under high pressure. Non-specific binding was blocked using 10% (v/v) goat serum (C0265; Beyotime). Next, the primary antibody (1:200) was applied per the manufacturer's instructions and incubated overnight at 4°C. The secondary antibody (1:200) was incubated for 30 min after washing on the next day. Signals were detected using the DAB Developer Kit (P0202, Beyotime). The stained sections were examined under a microscope (Leica Microsystems).

### Immunofluorescence

Kidney tissues and cells were fixed with paraformaldehyde. Samples were blocked with 10% (v/v) goat serum for 1 h. According to the instructions, the primary antibody (1:200) was added to cells or tissues and incubated overnight at 4°C. On the second day, antibodies labeled with fluorescence (1:200) were applied and incubated for 1 h. Nuclei were stained with 4′,6-diamidino-2-phenylindole (P0131, Beyotime). Sections were photographed using a fluorescence microscope (Leica Microsystems, Germany).

### Western blotting analysis

Protein samples were separated by sodium dodecyl sulfate (SDS)-polyacrylamide gel electrophoresis and transferred to a nitrocellulose membrane. The membranes were incubated with the primary antibodies overnight at 4 °C after blocking with 5% (w/v) skimmed milk for 2 h at room temperature. The next day, the membranes were blocked for 2 h using horseradish enzyme peroxidase-labelled secondary antibodies. Protein expression was detected using the ECL system (Yeasen, 36222ES76) and the Amersham Imager 600 System. Band intensities were quantified using ImageJ software.

### Cell culture

H.Y. Lan from the Chinese University of Hong Kong provided the human kidney TEC line (HK-2). The cells were maintained in DMEM/F12 medium (SH30023.FS, Hyclone) supplemented with 10% (v/v) fetal bovine serum (10100147C, Gibco) at 37°C with 5% CO_2_.

siRNA and overexpression plasmids were designed and constructed by Vigene Biosciences Inc. (Hanbio Biotechnology, China). The specific sequences are as follows: si-NC, 5’-UUCUCCGAACGUGUCACGUTT-3’, siACSF2-1, 5’-CAUUGUCAACAACUCCAACAUTT-3’, siACSF2-2, 5’-GCAUCUUAACAGCAAGACUGUTT-3’; siACSF2-2, 5’-CGAGGACUUCUUUCACACACATT-3’; siPGK1, and 5’-CAGUUGCUGUAGAACUCAATT-3’. Lipofectamine 2000 reagent (Invitrogen) was used to transfect the cells.

### Measurement of mitochondrial membrane potential (MMP)

To evaluate variations in MMP, anAn MMP assay kit with JC-1 (C2006, Beyotime) was employed to evaluate variations in MMP. Per manufacturer instructions, HK-2 cells were stained with JC-1 dye at 37°C for 20 min while keeping them in the dark. After washing with JC-1 buffer solution, the cells were observed under the fluorescence microscope (Leica Microsystems, Germany).

### Measurement of reactive oxygen species (ROS)

The intracellular ROS levels were measured using dihydroethidum (DHE, S0063, Beyotime) and dichlorodihydrofluorescein diacetate (DCFH-DA, S0033M, Beyotime). The cells were incubated in a cell culture medium containing 5 µM DHE or 10 µM DCFH-DA at 37°C 30 min. The labeled cells were observed under a fluorescence microscope (Leica Microsystems, Germany).

### Co-immunoprecipitation (Co-IP) and mass spectrometry

Total protein was extracted from the HK-2 cells. The collected supernatants were incubated overnight with the specified primary antibodies or IgG with gentle rotation at 4°C. Protein A/G agarose beads were added to capture the immune complexes, followed by incubation for 2 h at 4°C. Liquid chromatography-mass spectrometry (LC-MS/MS) was performed by OE Biotech (Shanghai, China).

### Statistical analysis

Normally distributed variables are presented as the mean ± standard deviation and were compared using Student’s t-test (for two groups) or one-way ANOVA with post hoc Tukey’s test (for multiple groups). Non-normally distributed variables are expressed as the median and interquartile range, and were analyzed using the Mann–Whitney U test (for two groups) or Kruskal–Wallis test (for multiple groups). Categorical variables were compared using the Chi-square test. Correlations between parameters were assessed using Pearson’s correlation analysis for normally distributed data and Spearman’s rank correlation analysis for non-normally distributed data. Statistical significance was set at *P* < 0.05. All data were analyzed using SPSS version 23.0.

## Results

### Ferroptosis-related ACSF2 is identified as one crucial DEG related to DN

**Figure 1.**
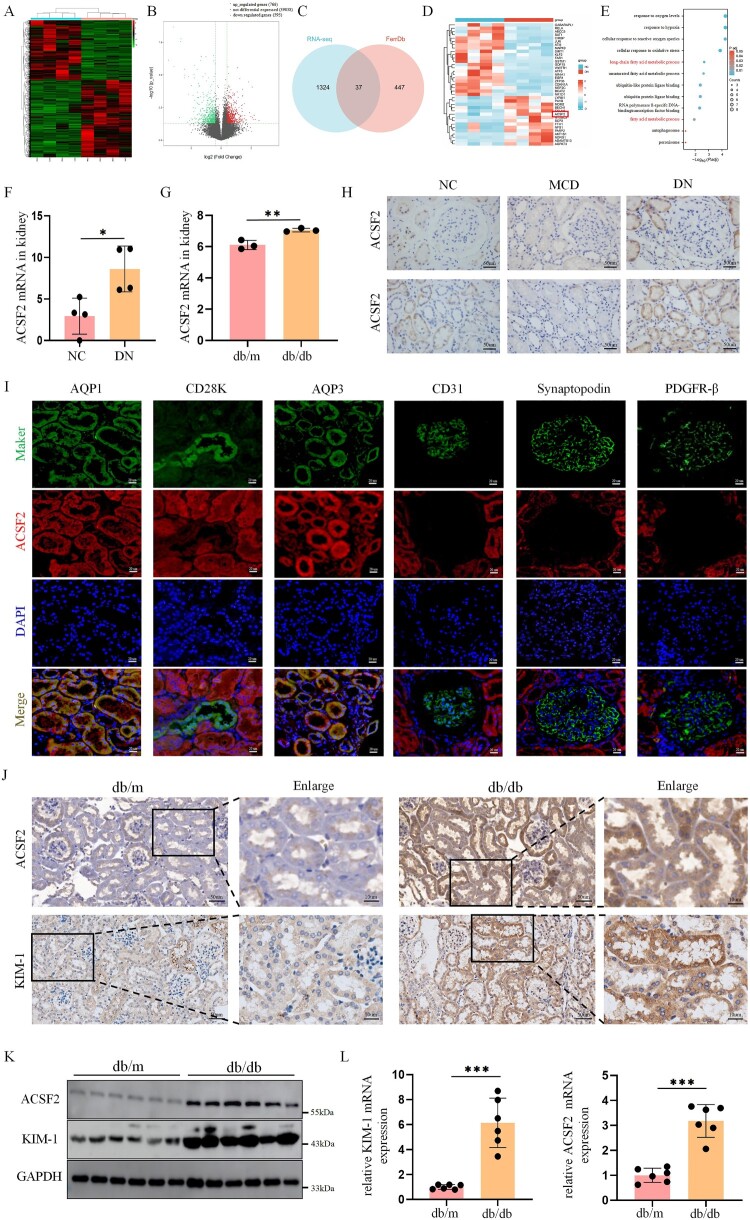
ACSF2 was significantly upregulated in the renal tubule of patients with DN and db/db mice. (A, B) Heatmaps of transcript and volcano map expression analysis of RNA-seq in kidney tissues of patients with DN (n = 4) and normal controls (n = 4). (C) Venn diagram showing the intersection of differentially expressed genes (DEGs) in our data and ferroptosis genes. (D) Heatmaps of the ferroptosis-related genes in our data. (E) GO and KEGG pathway enrichment analysis of the ferroptosis-related genes. (F) ACSF2 expression in the kidney of patients with DN (n = 4) and normal controls (n = 4). (G) ACSF2 expression in the kidney of db/db mice (n = 3) and db/m mice (n = 3) from the GEO database (GSE87359). (H) Representative photographs of ACSF2 immunohistochemical staining in patients with DN (n = 85), MCD patients (n = 35), and normal controls (n = 21) (scale bar = 50 µm). (I) Immunofluorescence double staining for ACSF2 and specific tubular/glomerular markers in renal specimens of patients with DN. ACSF2 was marked with Alexa-Fluor 594 conjugated secondary antibody. The proximal and distal tubular epithelial cells and collecting duct cells were marked as AQP1, CD28k and APQ3 with Alexa-Fluor 488 conjugated secondary antibody, respectively. Glomerular endothelial cells, podocytes and mesangial cells were marked as CD31, synaptopodin and PDGFR-β with Alexa-Fluor 488 conjugated secondary antibody, respectively (scale bar = 50 µm). (J) Representative photographs and relative quantification of ACSF2 and KIM-1 immunohistochemical staining in the murine kidney (n = 6) (scale bar = 50 µm, enlarged scale bar = 10 µm). (K, L) The level of ACSF2 and KIM-1 expression in renal tissue homogenate from db/db and db/m mice was determined by western blot analysis and qRT-PCR. The data represent the mean ± SEM. *p < 0.05; **p < 0.01, ***p < 0.001. NC, normal control; DN, diabetic nephropathy; MCD, minimal change disease.

First, RNA-seq was used to identify DEG transcripts in the renal tissues of patients with DN. Transcripts (1,361) linked to 1165 genes were identified as differentially expressed, based on a threshold of |log2FC| > 0.585 and an adjusted *P* value < 0.05, including 595 downregulated and 766 upregulated transcripts. The results are shown by heatmaps and the volcano plot (Figure 1(A,B)). The DEGs were overlapped with ferroptosis-related genes from the “FerrDb” database. The Venn diagram shows 37 overlapping genes (Figure 1(C,D)). Of these, 20 were driver genes, 16 were suppressor genes, and one was a marker gene (Table S2). To further validate the reliability of our RNA-seq data, we examined data from the GEO database were examined (GSE87359). Notably, only ACSF2 overlapped between the two datasets (Figure S1A). The results demonstrated that the expression of ACSF2 is upregulated in the renal tissues of both DN patients and db/db mice (Figure 1(F,G)). Enrichment analyses of the GO and KEGG pathways were conducted for the 37 ferroptosis-related genes (Figure 1(E)). The results showed that ACSF2 is enriched in various biological processes and pathways, including long-chain fatty acids and fatty acid metabolic processes, which have been proven important in ferroptosis. These findings indicated that ACSF2 may exacerbate DN progression by regulating ferroptosis.

To explore ACSF2's role in DN, ACSF2 protein expression levels were detected by immunohistochemistry in biopsy tissues of 83 patients with DN and type 2 diabetes and 56 controls (including 35 MCD and 21 renal cancer paracancerous tissues). ACSF2 expression was significantly elevated compared with that in normal controls and patients with MCD (*p* < 0.001 and *p* < 0.001, respectively) (Figure 1(H), S1B). ACSF2 was predominantly detected in the renal tubules, especially in the renal tubular epithelial cells (Figure 1(I)). Furthermore, our findings were validated in type 2 diabetic db/db mice (Figure 1(J–L), S1C, D)

### The expression of renal ACSF2 in patients with DN was correlated with renal tubule injury and ferroptosis

**Figure 2.**
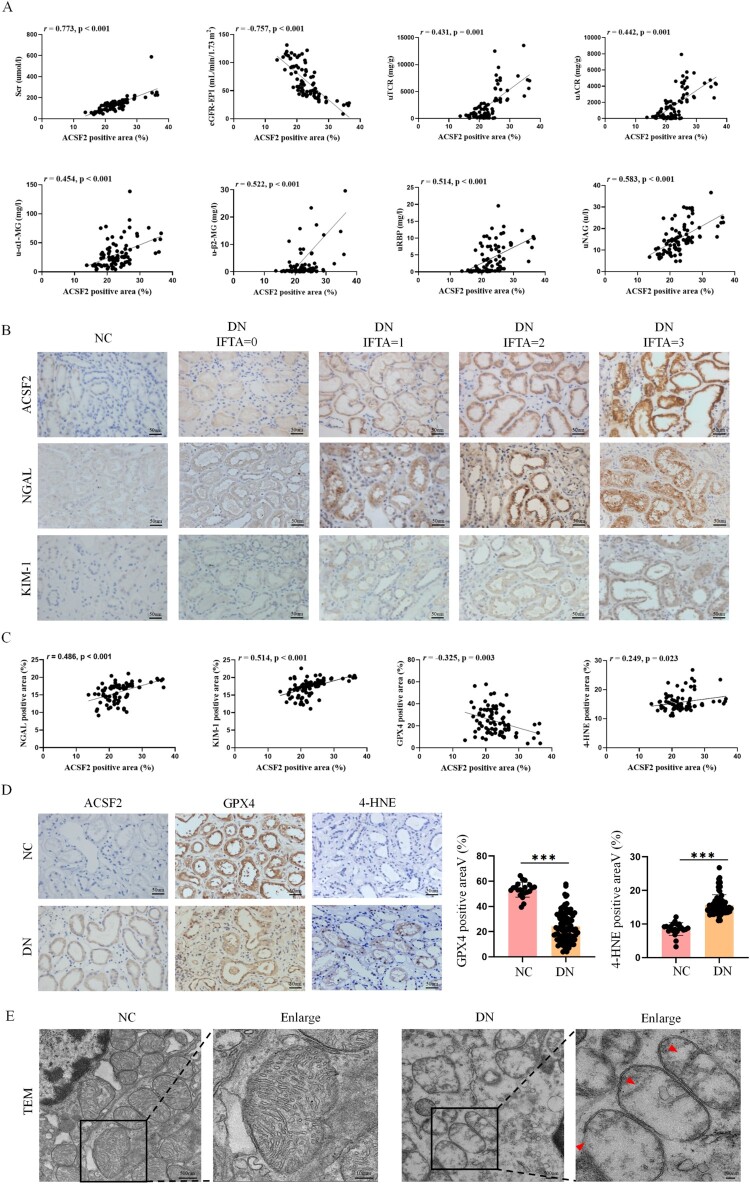
The expression of ACSF2 was correlated with renal tubule injury and ferroptosis. (A) Correlation analysis between ACSF2 and clinicopathological parameters of patients with DN. (B) Representative photographs of ACSF2, NGAL and KIM-1 immunohistochemical staining in patients with DN with different IFTA scores (scale bar = 50 µm). (C) Correlation analysis between ACSF2 and NGAL, KIM-1, GPX4 and 4-HNE in patients with DN. (D) Representative photographs and quantification of ACSF2, GPX4 and 4-NHE immunohistochemical staining in normal controls and patients with DN (scale bar = 50 µm). (E) TEM was used to analyze the representative micrographs of mitochondria in renal tubular epithelial cells of patients with DN. Arrows indicated damaged mitochondria (scale bar = 500 nm, enlarged scale bar = 100 nm). The data represent the mean ± SEM. *p < 0.05; **p < 0.01, ***p < 0.001. NC, normal control; DN, diabetic nephropathy; IFTA, interstitial fibrosis and tubular atrophy; TEM, transmission electron microscopy.

Next, we investigated the clinical significance of the ACSF2 levels in patients with DN. ACSF2 expression was positively correlated with serum creatinine and urine protein-creatinine ratio and negatively correlated with estimated glomerular filtration rate (eGFR). Also, ACSF2 expression was found to be positively correlated with markers of kidney tubular function (Figure 2(A)).

To further elucidate the potential correlation between ACSF2 and tubular injury progression in DN, patients with DN were classified based on interstitial fibrosis and tubular atrophy (IFTA) into four grades: no IFTA (fibrotic area = 0, IFTA = 0), mild IFTA (fibrotic area < 25%, IFTA = 1), moderate IFTA (fibrotic area 26-50%, IFTA = 2), and severe IFTA (fibrotic area > 50%, IFTA = 3) [17]. The baseline patient characteristics are shown in Table S3. With pathological injury aggravation in the renal tubule tissue, ACSF2 expression gradually increased (Figure 2(B), S2A). Significant NGAL and KIM-1 upregulation was also observed in the renal tubules of patients with DN and was positively correlated with ACSF2 expression (Figure 2(B,C)). Receiver operating characteristic curve analysis showed that ACSF2 was a good indicator for determining patients with DN and MCD or normal controls (AUC: 0.862, 96.77 sensitivity, 67.31% specificity) (Figure S2B). This indicates that ACSF2 plays an important role in renal tubule injury of patients with DN.

To determine the possible role of ACSF2 in DN, the expression of the ferroptosis marker GPX4 in the renal tissues of patients with DN was analyzed. GPX4 expression was significantly lower in patients with DN than in normal controls. We also observed that 4-HNE, a marker of oxidative stress-induced lipid peroxidation (LPO) and cytotoxicity, showed increased positive staining in patients with DN (Figure 2(D)). TEM revealed that the renal tubules of patients with DN exhibited pronounced mitochondrial damage, including mitochondrial swelling, reduced number of cristae, and matrix vacuolation (Figure 2(E)). This demonstrates ferroptosis of tubular epithelial cells in patients with DN. Furthermore, linear regression analysis revealed that ACSF2 negatively correlated with GPX4 expression and positively correlated with 4-HNE expression (Figure 2(C)).

Considering the association between ACSF2 expression in patients with DN and renal tubule injury and ferroptosis, we speculated that ACSF2 promotes tubular LPO and ferroptosis in DN.

### ACSF2 promoted ferroptosis by oxidative stress-induced lipid peroxidation in renal tubule of db/db mice

**Figure 3.**
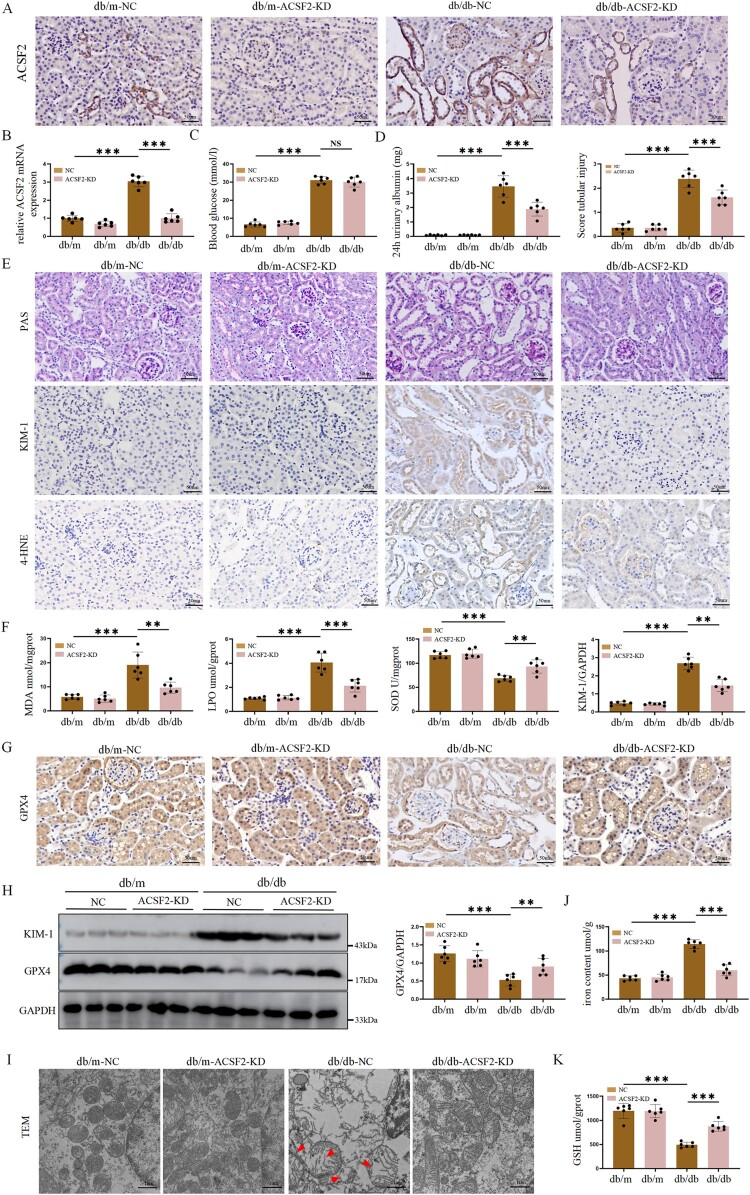
Silencing ACSF2 in vivo alleviated oxidative stress and ferroptosis in renal tubule of db/db mice. (A) Representative immunohistochemical staining of ACSF2 in different groups of db/db mice (scale bar = 50 µm). (B) qRT-PCR showing ASCF2 gene knockdown in the kidney. (C, D) Results of blood glucose and mouse urine albumin ELISA in different groups of db/db mice. (E) PAS staining detecting tubular injury and immunohistochemical of KIM-1 and 4-HNE in different groups of db/db mice (scale bar = 50 µm). (F) The levels of MDA, LPO and SOD in renal tissue of different groups of db/db mice. (G) Representative immunohistochemical staining of GPX4 in different groups of db/db mice (scale bar = 50 µm). (H) Western blot analysis and quantification of KIM-1 and GPX4 in different groups of db/db mice. (I) TEM was used to analyze the mitochondrial morphology in different groups of db/db mice, arrows indicated damaged mitochondria (scale bar = 1 µm). (J-K) The levels of iron content and GSH in renal tissue of different groups of db/db mice. Data represent the mean ± SEM for 6 mice. NC, AAV9-scramble. TEM, transmission electron microscopy.

We further investigatedFurthermore, ACSF2's role in tubular injury in vivo was investigated. We silenced ACSF2 in db/db mice was silenced using an AAV9-packaged ACSF2 knockdown plasmid containing the human renal tubular-specific KSP-cadherin gene promoter. These results confirmed that ACSF2 expression was significantly reduced in the renal tubules of AAV9-shACSF2 db/db mice (Figure 3(A,B); Figure S3A, B). No significant difference was observed in blood glucose levels in db/db mice after silencing ACSF2 expression (Figure 3(C)). However, the 24-h urinary albumin levels significantly decreased (Figure 3(D)). PAS staining revealed that ACSF2 deficiency attenuated renal tubular injury (Figure 3(E)). Furthermore, our this study’s results showed that KIM-1 levels decreased in db/db mice after silencing ACSF2 (Figure 3(E), G; Fig. S3C).

Next, we investigated whether ACSF2 affected oxidative stress was investigated. The levels of lipid peroxide 4-HNE in the kidney were significantly reduced in db/db mice after silencing ACSF2 (Figure 3(E)). Moreover, the concentrations of MDA and LPO were upregulated, and SOD levels were downregulated in db/db mice. However, these effects were reversed upon silencing ACSF2 (Figure 3(F)). LPO is a key ferroptosis feature. ACSF2 knockdown effect on oxidative stress-induced ferroptosis was also examined. The results showed that GPX4 levels increased in db/db mice after silencing ACSF2 (Figure 3(G), H; Figure S3C). Furthermore, renal tissues were examined using TEM. The results showed that db/db mice exhibited mitochondrial characteristics of ferroptosis, including mitochondrial cristae reduction and disappearance, rupture of the outer mitochondrial membrane, and vacuolization of mitochondria. The mitochondrial morphological changes in db/db mice were mitigated after silencing ACSF2 (Figure 3(I)). Consistently, iron levels were upregulated, and GSH levels were downregulated in db/db mice. However, these effects were reversed by silencing ACSF2 (Figure 3(J,K)). These findings indicated that ACSF2 promotes ferroptosis via oxidative stress-induced LPO in the renal tubules of db/db mice.

### Silencing ACSF2 alleviated renal tubule injury by reducing oxidative stress-induced ferroptosis.

**Figure 4.**
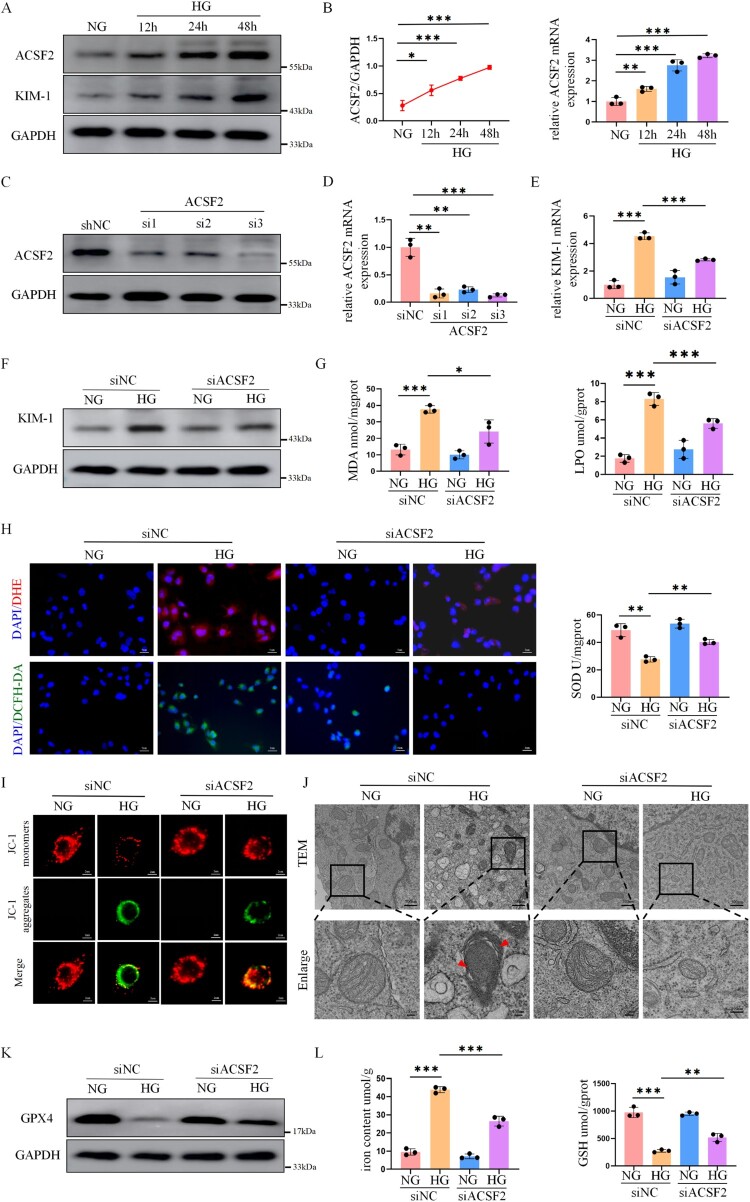
Silencing ACSF2 alleviated oxidative stress-induced ferroptosis in HG-stimulated HK-2 cells. (A, B) Western blot analysis and qRT-PCR of ACSF2 and KIM-1in HK-2 cells after exposure to HG at various time points. (C, D) Western blot analysis and RT-PCR were performed to detect the three siRNA silencing effects of ACSF2. (E, F) Western blot analysis and qRT-PCR of KIM-1 in HK-2 cells with ACSF2 silencing. (G) The levels of MDA, LPO and SOD in HK-2 cells. (H) Representative image of DHE and DCFH-DA staining in HK-2 cells (scale bar = 5 µm). (I) HK-2 cells were labeled with fluorescent probe JC-1 to evaluate MMP changes in situ by fluorescence microscope. J-aggregate, red; JC-1 monomer, green (scale bar = 2 µm). (J) TEM was used to analyze the mitochondrial morphology in HK-2 cells, and the arrows indicated damaged mitochondria (scale bar = 500 nm, enlarged scale bar = 100 nm). (K) Western blot analysis of GPX4 in HK-2 cells with ACSF2 silencing. (L) The iron and GSH contents in HK-2 cells. The data represent the mean ± SEM. *p < 0.05; **p < 0.01, ***p < 0.001. NG, normal glucose, 5.5 mmol/L; HG, high glucose, 30 mmol/L; TEM, transmission electron microscopy.

To further investigate ACSF2 role in ferroptosis, HK-2 cells were exposed to normal high glucose (30 mM) at different time points. The results indicated that ACSF2 expression significantly increased over time (Figure 4(A,B), and S4A). Simultaneously, KIM-1 expression significantly increased, indicating the effective induction of HK-2 cell injury (Figure 4(A)). However, mannitol had no effect on ACSF2 expression (Figure S4B). We then generated three different siRNAs targeting ACSF2, and the knockdown efficiency was confirmed by western blotting and qRT-PCR (Figure 4(C,D) and S4C). The siACSF2-3 was used for subsequent experiments. Silencing ACSF2 significantly reduced KIM-1 expression following high-glucose (HG) stimulation (Figure4(E,F); Figure S4D). Further, the effect of silencing ACSF2 on oxidative stress was investigated in vitro. Our findings showed that ACSF2 silencing significantly reduced MDA and LPO levels and increased SOD activity (Figure 4(G)). Subsequently, we observed that ACSF2 silencing demonstrated a notable decrease in the relative fluorescence intensity of DHE and DCFH-DA compared to the control group (Figure 4(H)). In addition, mitochondrial peroxide levels were assessed using the mitochondria-targeted fluorescent probe MitoNeoD. Our results showed that high glucose stimulation significantly increased mitochondrial ROS production in HK-2 cells, whereas ACSF2 silencing markedly reduced mitochondrial ROS levels under high glucose conditions (Figure S4F). Notably, the mitochondrial membrane potential of HK-2 cells was assessed using a JC-1 probe. The results showed that the green fluorescence intensity increased in HG-treated HK-2 cells compared with the NC group, and these changes could be reversed by silencing ACSF2 (Figure 4(I)).

Consistent with the previously reported morphological features of mitochondria during ferroptosis, TEM showed that some mitochondria in the HG group had reduced mitochondrial cristae structures, vacuolation, and incomplete membranes. However, silencing ACSF2 significantly reduced ferroptosis-related mitochondrial damage induced by HG (Figure 4(J)). In addition, our results showed that silencing ACSF2 significantly restored GPX4 expression following HG stimulation (Figure 4(K), and S4E). Moreover, silencing ACSF2 significantly reduced ferroptotic events, including decreased iron content and increased GSH levels (Figure 4(L)). In addition, silencing ACSF2 inhibited erastin-induced ferroptosis in HK-2 cells (Figure S4G).

These results suggest that silencing ACSF2 in HK-2 cells notably decreases oxidative stress and LPO levels and mitochondrial damage associated with ferroptosis caused by HG.

### ACSF2 modulates the Keap1/Nrf2 signaling through its regulation of PGK1 phosphorylation

**Figure 5.**
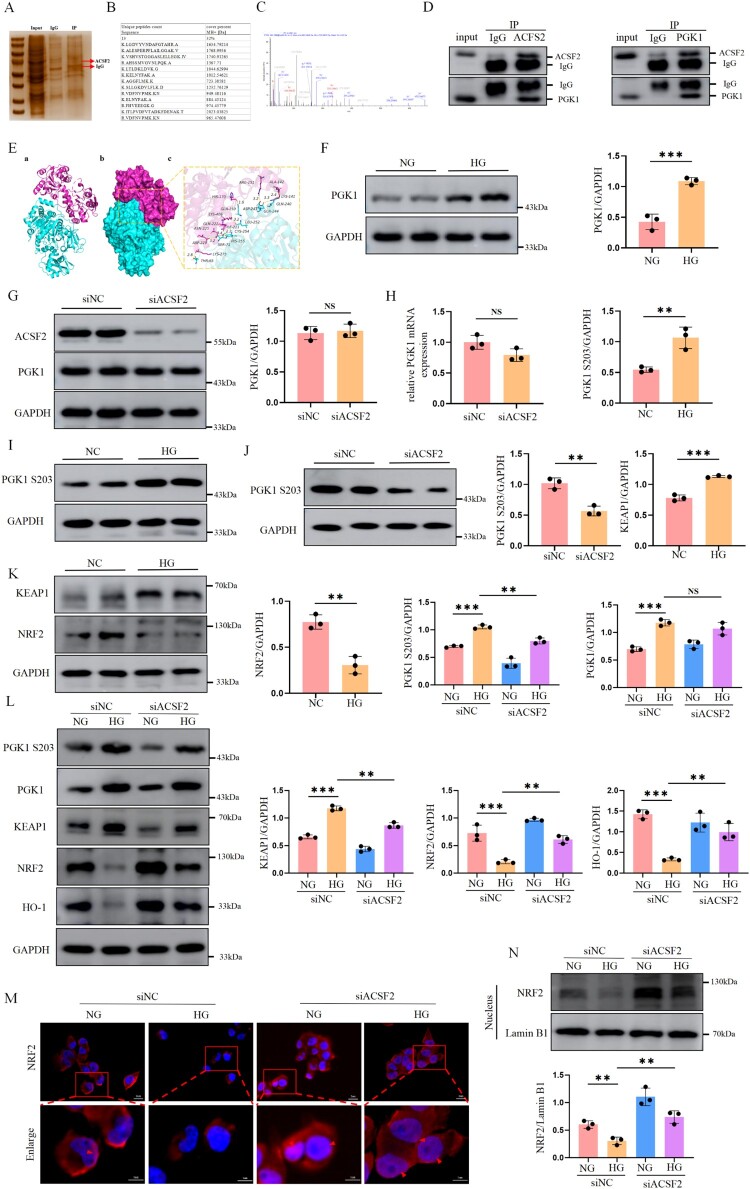
Silencing ACSF2 promoted Nrf2 expression and nuclear translocation by inhibiting PGK1 phosphorylation. (A) Proteins that interacted with ACSF2 were detected by silver staining SDS-PAGE gel and mass spectrometry. (B) The peptide segment for the PGK1 fragment identified by LC-MS/MS. (C) Representative MS spectra for PGK1 fragment identified by LC-MS/MS. (D) Total protein samples from HK-2 cells were extracted, and interacting ACSF2 proteins and PGK1 were detected using Co-IP assays followed by western blotting. (E) The binding mode of the complex PGK1 with ACSF2. (a) The backbone of protein was rendered in tube and colored in cyan (ACSF2) and red (PGK1). (b) PGK1 and ACSF2 protein is rendered by the surface. (c) The detail binding mode of PGK1 with ACSF2. Yellow dash represents hydrogen bond or salt bridge. (F) Western blot analysis and quantification of PGK1 in HG-induced HK-2 cells. (G) Western blot analysis and quantification of ACSF2 and PGK1 in HK-2 cells with ACSF2 silencing. (I) Western blot analysis and quantification of phosphorylation of PGK1 S203 in HG-induced HK-2 cells. (J) Western blot analysis and quantification of phosphorylation of PGK1 S203 in HK-2 cells with ACSF2 silencing. (K) Western blot analysis and quantification of Keap1 and Nrf2 in HG-induced HK-2 cells. (L) Western blot analysis and quantification of phosphorylation of PGK1 S203, PGK1, Keap1, Nrf2 and HO-1 in HK-2 under different treatments. (M, N) The expression of nuclear Nrf2 was evaluated by immunofluorescence and western blot analysis (scale bar = 5 µm, enlarged scale bar = 1 µm). The data represent the mean ± SEM. *p < 0.05; **p < 0.01, ***p < 0.001. HG, high glucose, 30 mmol/L; NG, normal glucose, 5.5 mmol/L. ns, not significant.

To examine the mechanism responsible for ACSF2 activity in HK-2 cells, liquid chromatography-tandem mass spectrometry (LC-MS/MS) analysis was performed on interacting ACSF2 proteins. Silver staining revealed that ACSF2 was effectively pulled down from IP cell lysates and was subjected to LC-MS analysis (Figure 5(A)). The result identified 572 potentially interacting proteins. We identified a candidate interacting protein, PGK1. According to the mass spectrometry results, 13 unique PGK1 peptides (cover rate = 32%) were enriched (Figure 5(B,C)). Co-IP assays were used to confirm the results of mass spectrometry analysis, which revealed that ACSF2 pulled down PGK1. The reverse Co-IP revealed that ACSF2 was significantly precipitated by PGK1 in HK-2 cells (Figure 5(D)). The co-localization assay revealed that ACSF2 and PGK1 were co-located in the cytoplasm of HK2 cells, providing evidence for their interaction (Figure S5A). Furthermore, we used the HDOCK tool to perform molecular docking of ACSF2 and PGK1, with results indicating a stable docking and −237.00 kcal/mol binding energy (Figure 5(E)). Our data indicate that ACSF2 interacts with PGK1.

Western blotting showed that PGK1 expression was higher in HG-treated HK-2 cells than in those treated with NG (Figure 5(F)). Next, we used western blotting and qRT-PCR to examine PGK1 protein and mRNA levels in HK-2 cells with ACSF2 silencing. The results showed no significant difference in PGK1 protein and mRNA levels (Figure 5(G,H)). Studies have revealed that PGK-1 post-translational modifications, particularly phosphorylation, play crucial roles in normal and disease-related processes [18,19]. Protein structural analysis revealed that the S203 PGK1 residue was close to the binding sites of the two proteins (Figure 5(E)). Western blotting showed that PGK1 S203 expression increased in HK-2 cells under HG conditions, whereas ACSF2 silencing significantly inhibited PGK1 S203 phosphorylation in HK-2 cells (Figure 5(I,J)). Studies have demonstrated that PGK1 may play a role in modulating the interaction between Keap1 and Nrf2 [20]. The Keap1/Nrf2 pathway is crucial for oxidative stress and ferroptosis. Therefore, we hypothesized that ACSF2 is involved in tubular epithelial ferroptosis in DN by regulating the Keap1/Nrf2 pathway via phosphorylated PGK1. To test this hypothesis, we first investigated the expression of the Keap1/Nrf2 signaling pathway under HG-stimulation in HK-2 cells. Western blotting showed that the Keap1 expression increased, whereas that of Nrf2 decreased in HK-2 cells after HG stimulation (Figure 5(K)), consistent with the findings of previous studies. Further studies revealed that ACSF2 silencing downregulated PGK1 S203 and Keap1 expression, restored Nrf2 and HO-1 expression, and promoted Nrf2 nuclear translocation (Figure 5(L–N)).

### PGK1 inhibits ACSF2 ubiquitination to improve the stability of the ACSF2 protein

**Figure 6.**
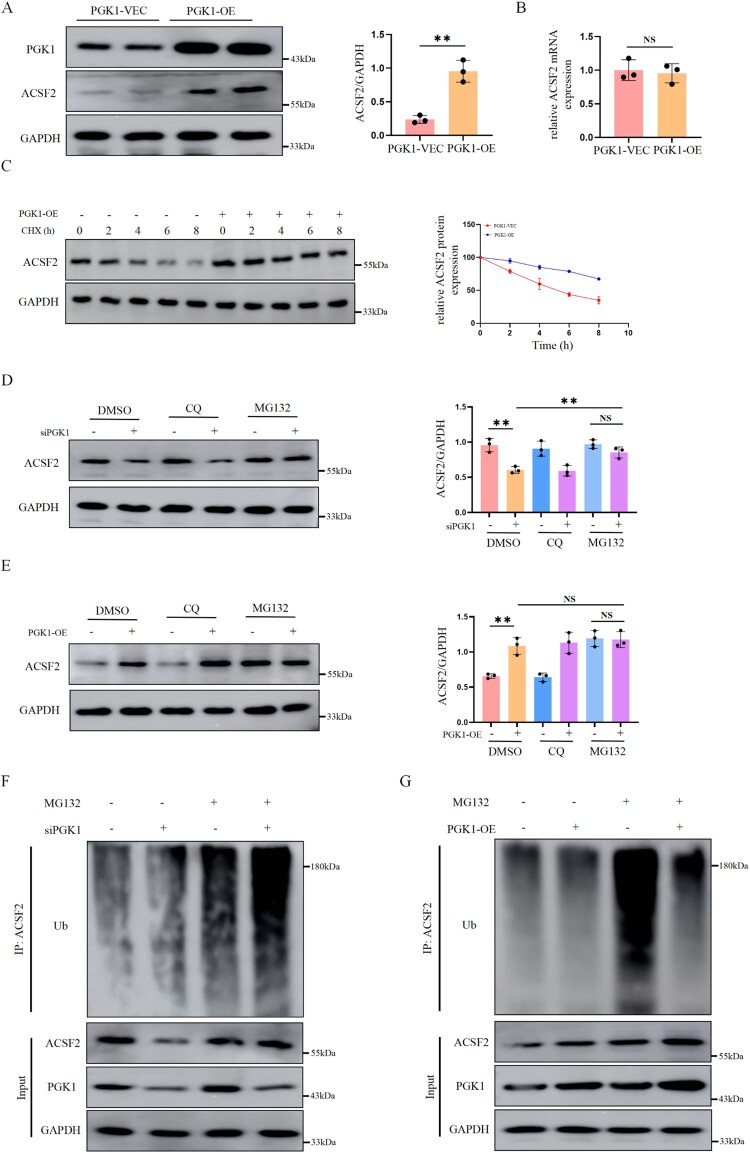
PGK1 inhibits ACSF2 ubiquitination and degradation. (A, B) Western blot analysis and qRT-PCR of ACSF2 in HK-2 cells with PGK1 overexpression. (C) HK-2 cells were treated with cycloheximide (CHX, 100ug/ml) at different time points. Cell lysates were harvested and analyzed using western blotting. (D, E) The protein level of ACSF2 in HK-2 cells with PGK1 silencing and PGK1 overexpression treated with MG132 (20 µg/mL) or chloroquine (CQ, 40 µg/mL) were analyzed by western blotting. (F, G) Impact of PGK1 silencing and PGK1 overexpression on ACSF2 ubiquitination in HK-2 cells. The data represent the mean ± SEM. *p < 0.05; **p < 0.01, ***p < 0.001. OE, overexpression; ns, not significant; CQ, chloroquine; ub, ubiquitination.

To explore the role of PGK1, we detected the expression of PGK1 was detected in HK-2 cells. We found that PGK1 overexpression significantly upregulated ACSF2 protein levels in HK-2 cells, whereas no statistically significant difference was observed in ACSF2 mRNA levels (Figure 6(A,B)). Therefore, we speculate that PGK1 enhances ACSF2 protein stability. Next, 100 µg/mL CHX was applied to HK-2 cells for different durations to prevent protein synthesis, and the degradation rates of the ACSF2 protein were determined by western blot. The data showed that HK-2 cells stably overexpressing PGK1 significantly reduced ACSF2 protein half-life (Figure 6(C)), suggesting that PGK1 affects ACSF2 protein degradation. Furthermore, we found that MG132, a proteasome inhibitor, but not the lysosomal inhibitor chloroquine, reversed the decrease in ACSF2 protein levels caused by PGK1 knockdown (Figure 6(D)). However, MG132 did not enhance the increase in ACSF2 protein levels induced by PGK1 overexpression (Figure 6(E)). Collectively, these results suggest that PGK1 upregulates ACSF2 expression by stabilizing the ACSF2 protein via the proteasome pathway.

Considering that PGK1 obstructs proteasomal ACSF2 degradation and that ubiquitination is the main pathway for proteasome-mediated protein degradation, we hypothesized that PGK1 is involved in ACSF2 ubiquitination. To test this hypothesis, a ubiquitination assay was conducted to investigate the PGK1 effect on post-translational ACSF2 modification. The results showed that endogenous ACSF2 ubiquitination levels increased in cells that underwent PGK1 knockdown, whereas endogenous ACSF2 ubiquitination levels significantly decreased in PGK1-overexpressing HK-2 cells compared with that in control cells (Figure 6(F,G)).

## Discussion

**Figure 7.**
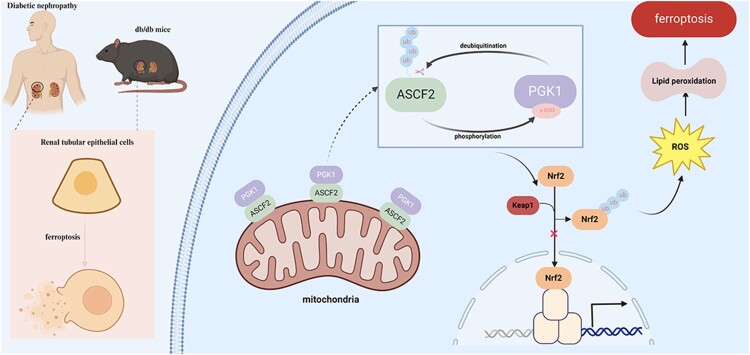
ACSF2-PGK1 interaction promotes ferroptosis in renal tubular epithelial cells of diabetic nephropathy by regulating Keap1/Nrf2 signaling.

In recent years, DN incidence has dramatically increased. However, current treatments remain inadequate [21,22]. Targeting the ferroptosis pathway offers a promising approach for developing novel therapeutic strategies for DN [23]. Our results revealed that the ferroptosis-related gene ACSF2 was overexpressed in patients with DN and was correlated with renal tubular injury. Moreover, ACSF2 was found to promote renal tubular injury by enhancing oxidative stress-induced ferroptosis. This mechanism has not been previously reported. Moreover, silencing ACSF2 decreased S203 phosphorylation in PGK1 cells and inhibited Keap1 expression. This results in increased Nrf2 expression and nuclear translocation, which, in turn, reduces ferroptosis. Further, PGK1 promotes ACSF2 stabilization, forming a positive feedback loop. Collectively, these findings indicated that ACSF2 is a promising therapeutic target for DN, highlighting its potential as a novel treatment strategy (Figure 7).

ACSF2 is a mitochondrial enzyme involved in fatty acid metabolism. They also play critical roles in lipid biosynthesis and energy production. Recently, ACSF2 biological role has received extensive attention [24]. Shi et al. reported that ACSF2 is a key factor in ferroptosis regulation in ulcerative colitis [14]. Another study showed that ACSF2 contributed to kidney damage by mediating mitophagy in HK-2 cells [11]. However, the precise mechanism of ACSF2 action in DN remains unclear. In this study, we discovered that ACSF2 expression was significantly elevated in patients with DN and db/db mice. ACSF2 was mainly located in the proximal renal tubules and positively correlated with renal tubule injury, suggesting that ACSF2 is a crucial factor in renal tubular injury in patients with DN. Furthermore, ACSF2 expression positively correlated with urinary protein and negatively correlated with eGFR, indicating its potential as a disease severity indicator in patients with clinical DN. These findings highlight the critical role of ACSF2, suggesting it could serve as a novel molecular target for DN diagnosis and treatment.

Recent studies have highlighted the critical role of tubular damage, suggesting that it plays a more central role in DN progression than previously thought [25,26]. Tubular cells are particularly vulnerable to metabolic stress, inflammation, and oxidative damage owing to their high mitochondrial content. The “glomerulocentric view” has long dominated DN understanding. However, increasing evidence suggests that renal tubular injury may precede glomerular injury. The proximal tubular dysfunction may contribute to the glomerulopathy through tubulo-glomerular crosstalk mechanisms [27,28]. Hasegawa et al. reported that renal tubular Sirt1 mitigates diabetic albuminuria by epigenetically suppressing the overexpression of Claudin-1 in podocytes [28]. Chen, et al. demonstrated that the expression of CNPY2 was increased in the renal tubules of patients with DN and db/db mice and the knockdown of CNPY2 alleviated renal tubule damage and reduced urinary albumin-to-creatinine ratio levels in db/db mice [29]. Based on these findings, we speculated that ACSF2 may influence albumin excretion primarily through mechanisms related to tubular dysfunction rather than direct glomerular injury.

Renal tubular injury triggered by ferroptosis is essential for DN pathogenesis [10,30]. Consistent with previous studies, we observed ferroptosis in the renal tubular epithelial cells of patients with DN. Moreover, ACSF2 expression positively correlated with LPO product (4-HNE) expression and negatively correlated with the expression of the ferroptosis marker (GPX4) in the renal tubules of patients with DN. This study revealed that the pathological changes associated with ferroptosis were significantly mitigated by silencing ACSF2 in HG-treated HK-2 cells and db/db mice. Furthermore, we found that silencing ACSF2 decreased MDA levels and 4-HNE expression and reduced HG-induced ROS accumulation. Increasing evidence suggests that LPO, driven by excessive oxidative stress, plays a key role in ferroptosis progression. The crosstalk between oxidative stress and ferroptosis has been observed in various diseases [31,32]. However, the interaction between oxidative stress and ferroptosis in DN remains unclear. Our data suggest that ACSF2 promotes ferroptosis through oxidative stress-induced LPO, which may be a key molecular mechanism underlying renal tubule injury in DN.

Moreover, using LC-MS/MS analysis, we identified a novel interacting protein, PGK1, that interacts with ACSF2 in HK-2 cells. PGK1 is the key enzyme involved in glycolysis. It is essential for regulating energy metabolism and preserving mitochondrial homeostasis [33,34]. Our data indicated that silencing ACSF2 did not alter PGK1 expression. However, considering that PGK1 activity is regulated by various post-translational modifications and based on protein spatial structure analysis, we discovered that the PGK1 S203 residue is situated close to the binding site of the two proteins. As expected, this study demonstrated that ACSF2 affects S203 phosphorylation in PGK1. Recent studies have shown that PGK1 negatively regulates Nrf2 expression. PGK1 inhibition or depletion leads to Keap1 dimerization, followed by Keap1-Nrf2 disassociation and Nrf2 signaling activation [35,36]. Xu et al. have reported that GIT1 regulates Keap1 dimerization by modulating PGK1 phosphorylation at S203, which subsequently reduces Nrf2 ubiquitination and degradation [20]. However, no study has reported PGK1 function in DN. In this study, we observed that PGK1 and PGK1 S203 expressions were upregulated in HG-treated HK-2 cells. Silencing ACSF2 inhibited PGK1 S203 phosphorylation, decreased Keap1 expression, and promoted Nrf2 expression and nuclear translocation. Increasing evidence indicates a strong link between ferroptosis and oxidative stress and highlights the essential role of Nrf2 in maintaining cellular redox balance and inhibiting ferroptosis [37,38]. This study provides additional evidence that ACSF2 couples oxidative stress and ferroptosis in renal tubules and reveals the possible mechanism of action of ACSF2 in DN.

Ubiquitination is an important post-translational modification that plays a crucial role in maintaining cellular protein homeostasis [39]. Increasing evidence suggests that ubiquitin modifications play a role in ferroptosis [40,41]. Studies have shown that PGK1 inhibits AR ubiquitination and increases AR stability [42]. In this study, PGK1 overexpression significantly upregulated ACSF2 expression. Further investigation showed that PGK1 promotes ACSF2 accumulation by inhibiting its ubiquitinated degradation.

In summary, this study demonstrated that ACSF2 was upregulated in patients with DN and that high ACSF2 expression was associated with renal tubule injury and disease severity. ACSF2-mediated PGK1 phosphorylation plays a crucial role in the ferroptosis of renal tubular epithelial cells by regulating Keap1 dimerization and Nrf2 ubiquitination. PGK1 promotes ACSF2 stabilization by regulating ACSF2 ubiquitination, thus forming a positive feedback loop. The ACSF2/PGK1 axis may be a potential therapeutic target in DN.

## Supplementary Material

Supplemental material_Original full length western blots.pdf

Supplemental_material_Figure_clean_version.pdf

Supplementary_data_Table_clean_version.pdf

## Data Availability

The data that support the findings of this study are available from the corresponding author, [Ling Jiang], upon reasonable request.
