# Supplementary material for: ACSF2-PGK1 interaction promotes ferroptosis in renal tubular epithelial cells of diabetic nephropathy by regulating Keap1/Nrf2 signaling
Source: Redox Rep. 2025 Jul 16;30(1):2529618. doi: 10.1080/13510002.2025.2529618 (PMC12269058; doi:10.1080/13510002.2025.2529618)
Supplement: Supplemental material_Original full length western blots.pdf [file YRER_A_2529618_SM9584.pdf]

Figure 1K

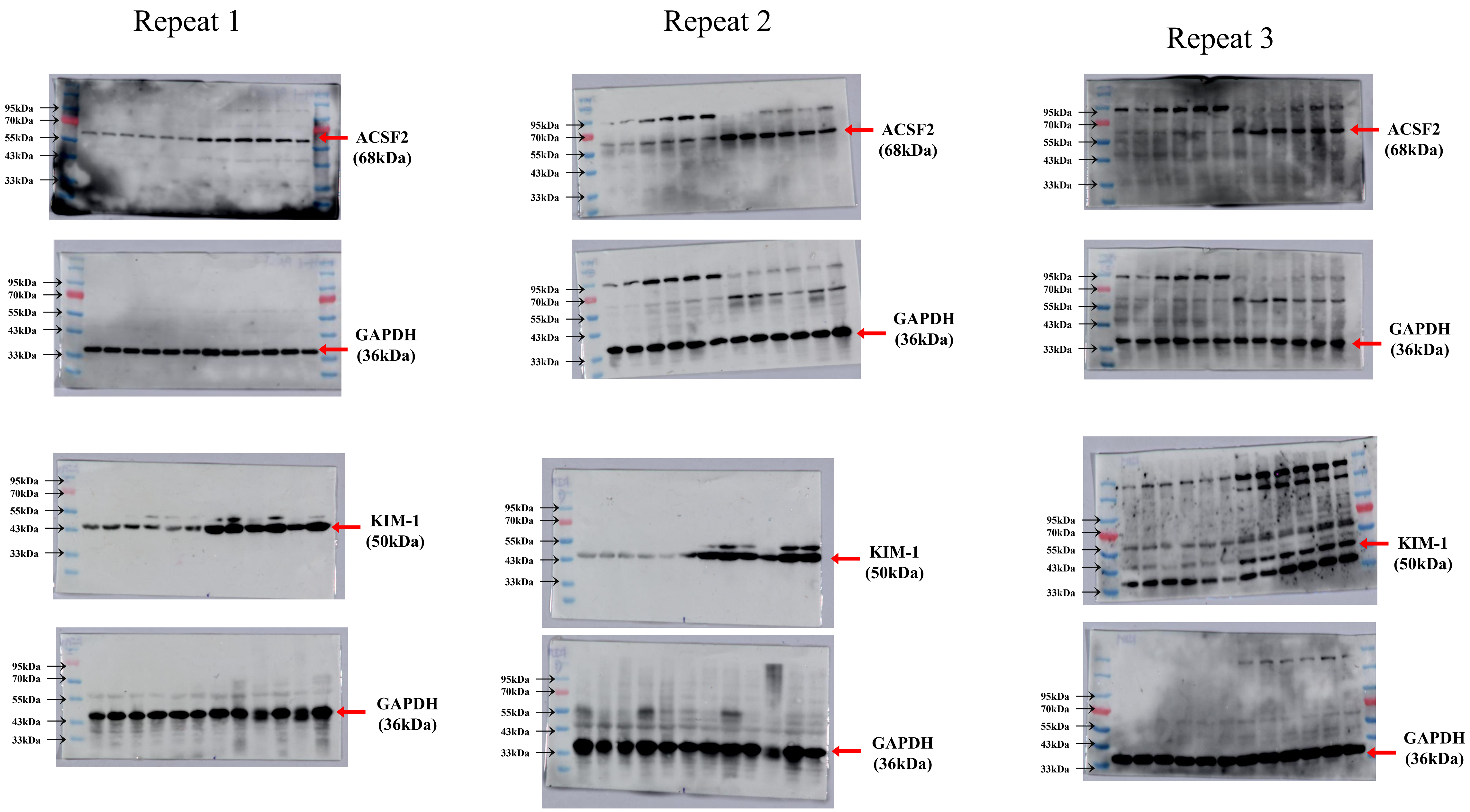
Repeat 1
Repeat 2
Repeat 3
95kDa
70kDa
55kDa
43kDa
33kDa
ACSF2
(68kDa)
GAPDH
(36kDa)
KIM-1
(50kDa)

Figure 3G

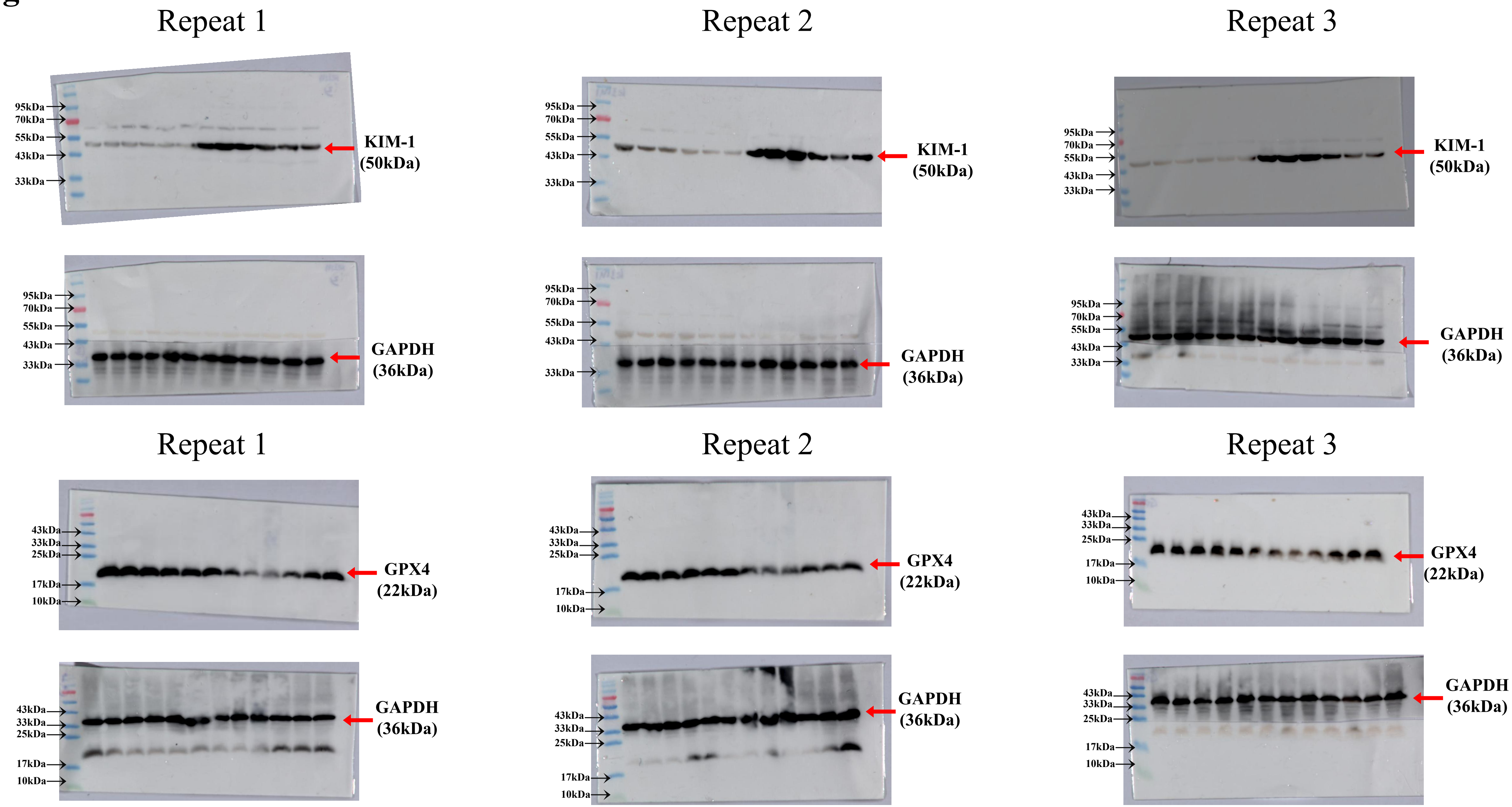
Repeat 1
Repeat 2
Repeat 3
95kDa
70kDa
55kDa
43kDa
33kDa
KIM-1
(50kDa)
GAPDH
(36kDa)
Repeat 1
Repeat 2
Repeat 3
43kDa
33kDa
25kDa
17kDa
10kDa
GPX4
(22kDa)
GAPDH
(36kDa)

Figure 4A

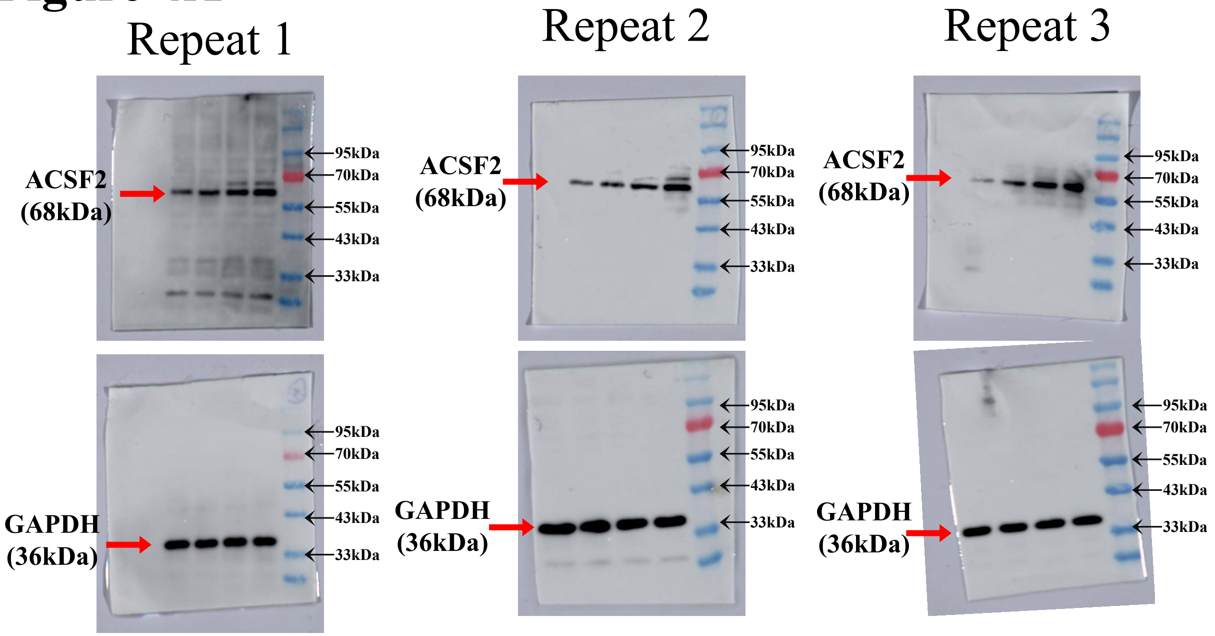

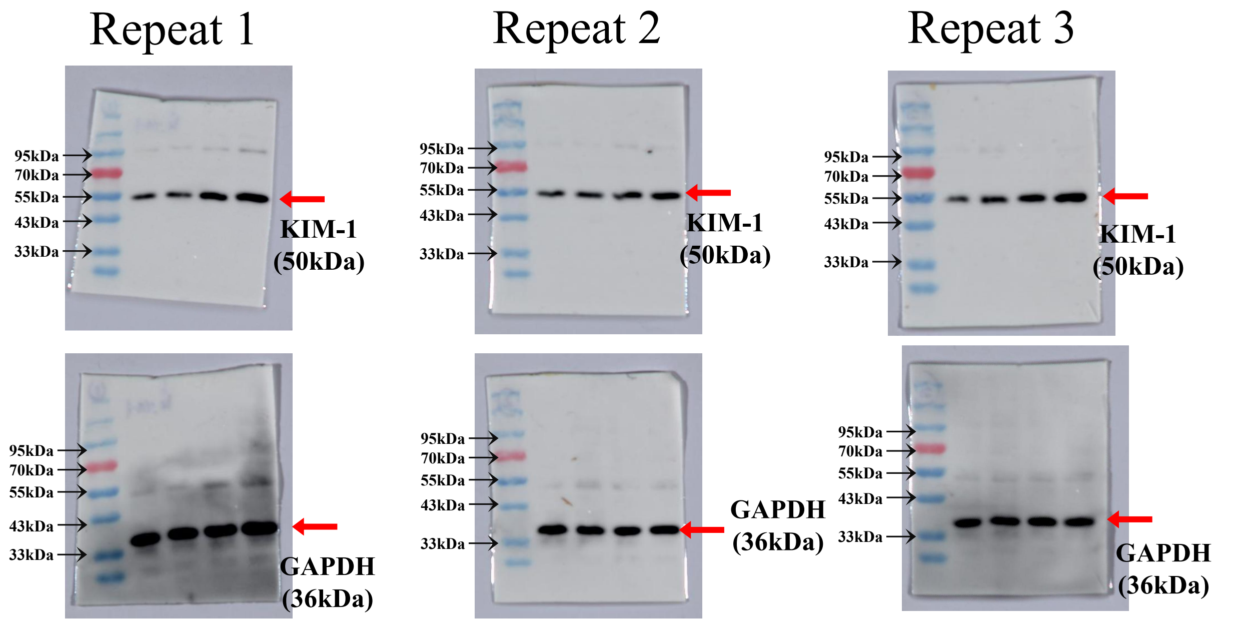

Figure 4C

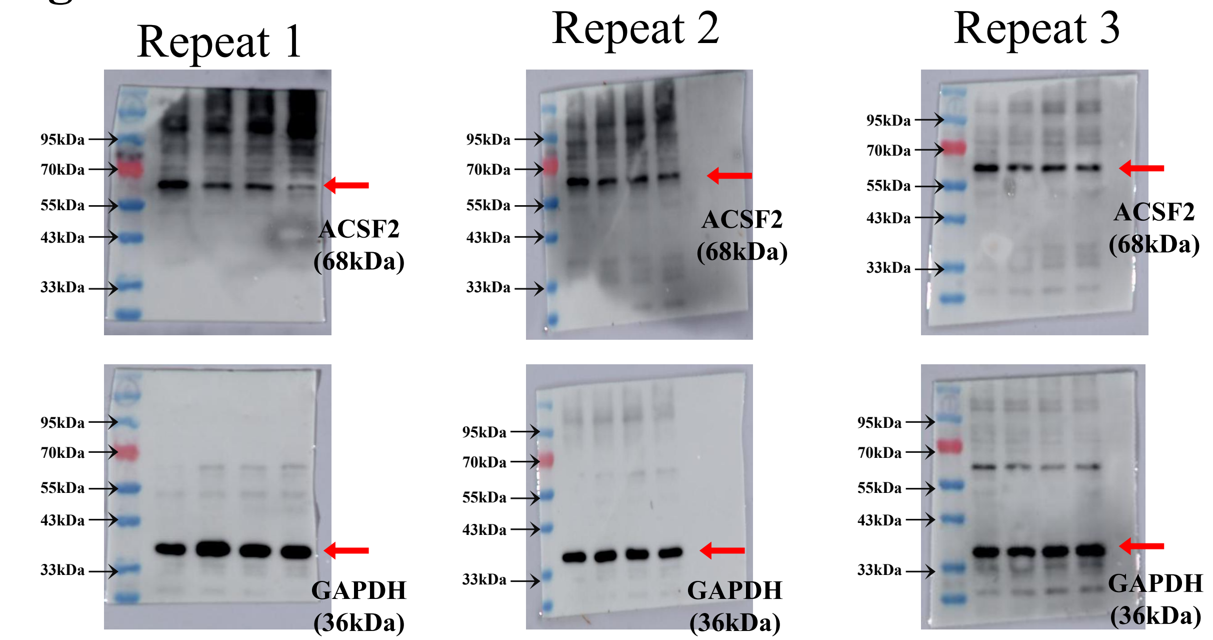

Figure 4E

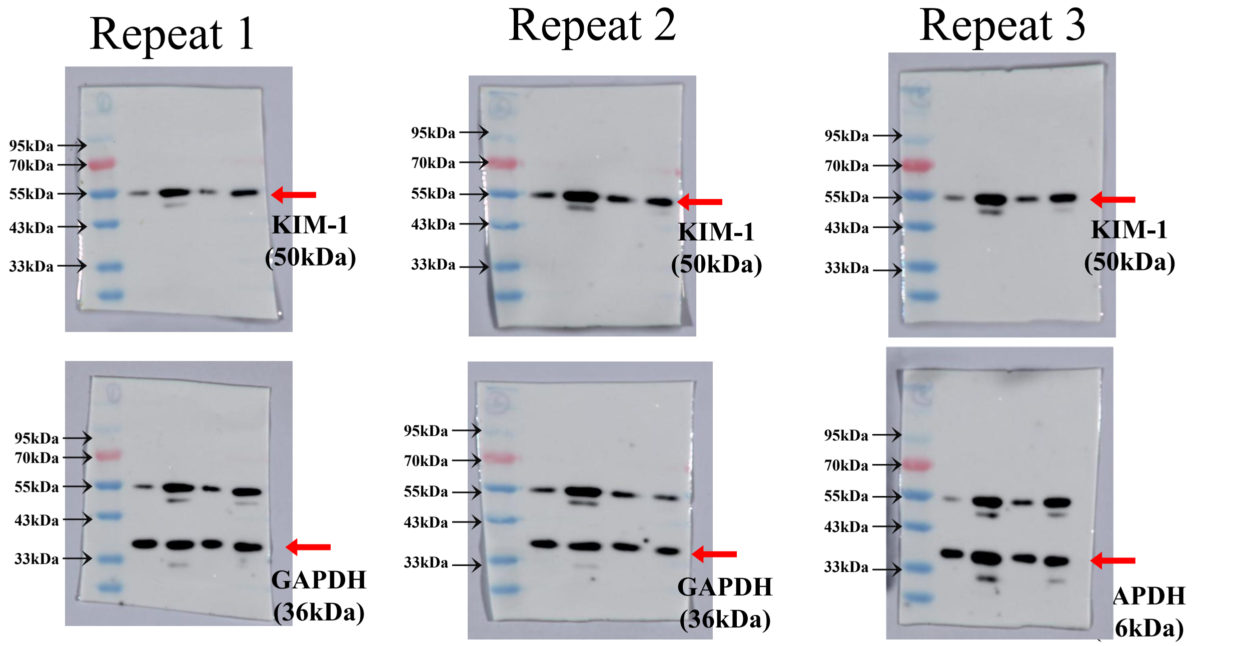

Figure 4J

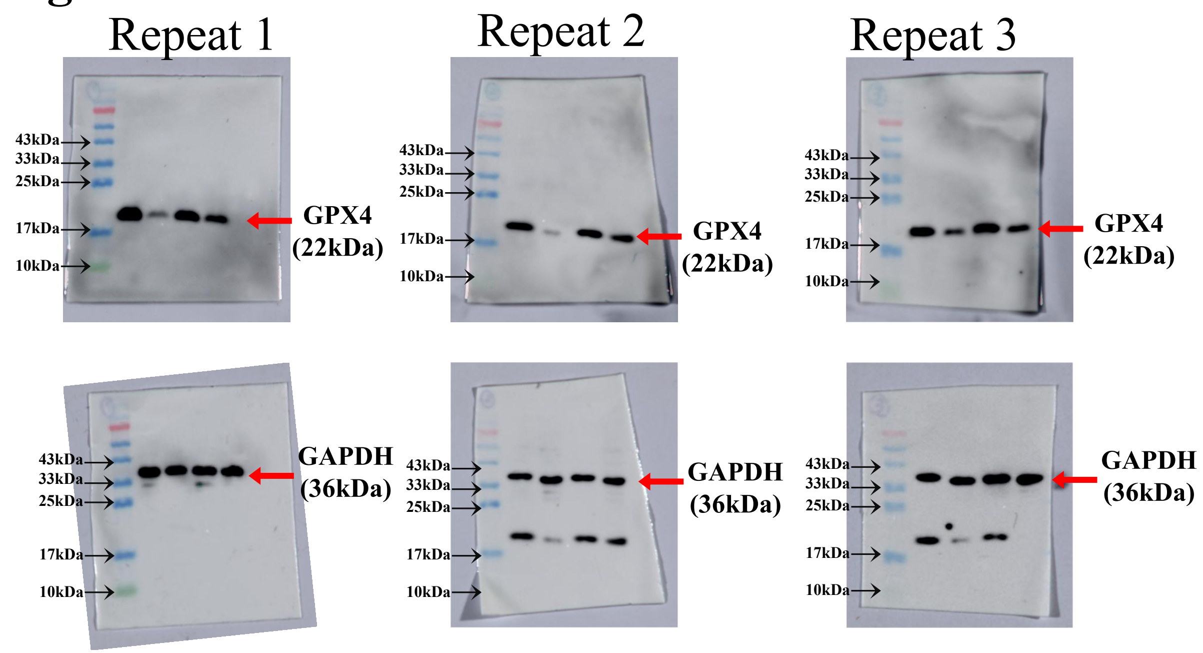

Figure 5F

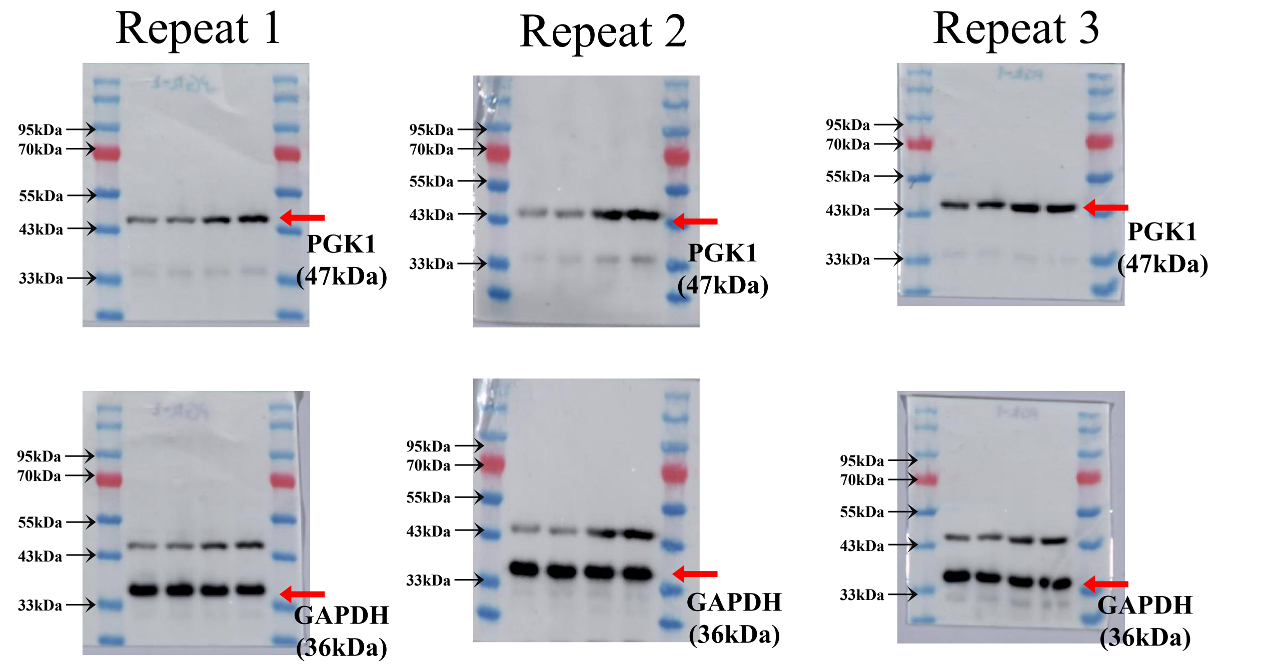

Figure 5G

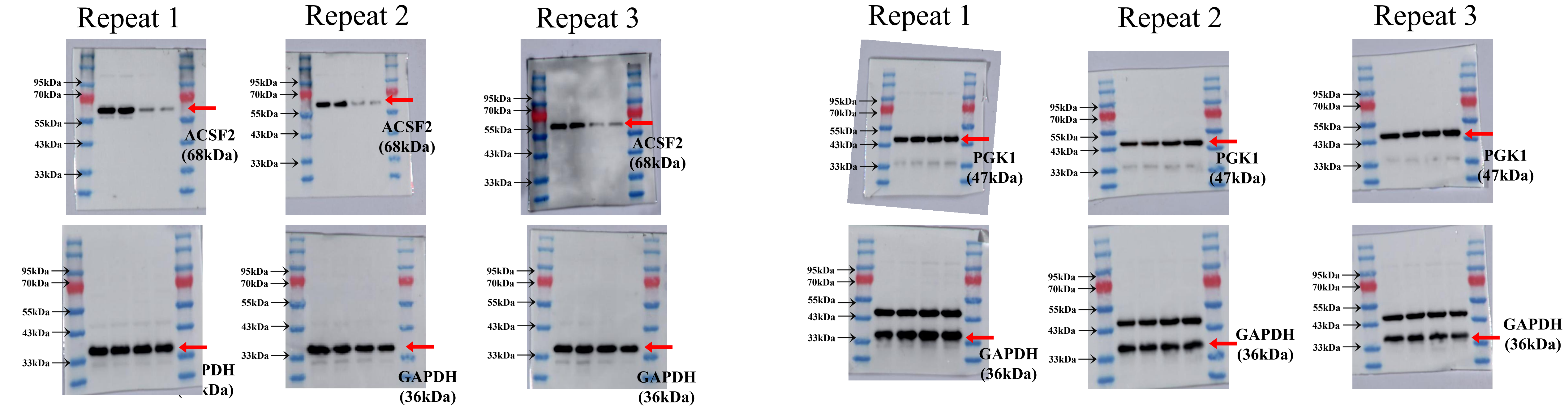

Figure 5I

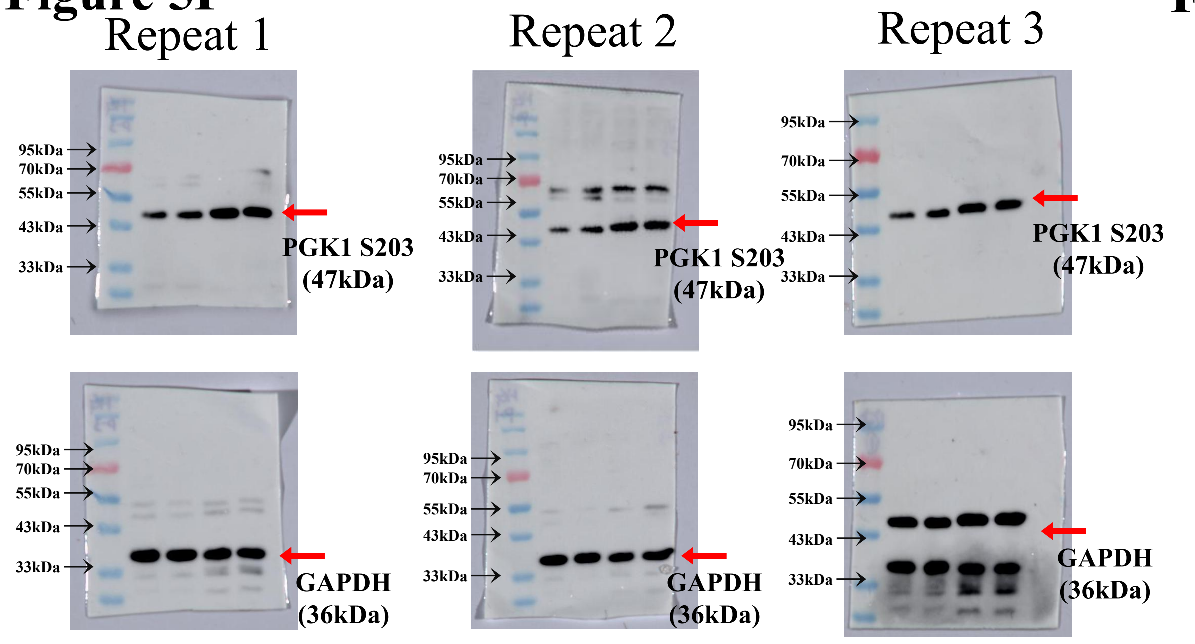

Figure 5J

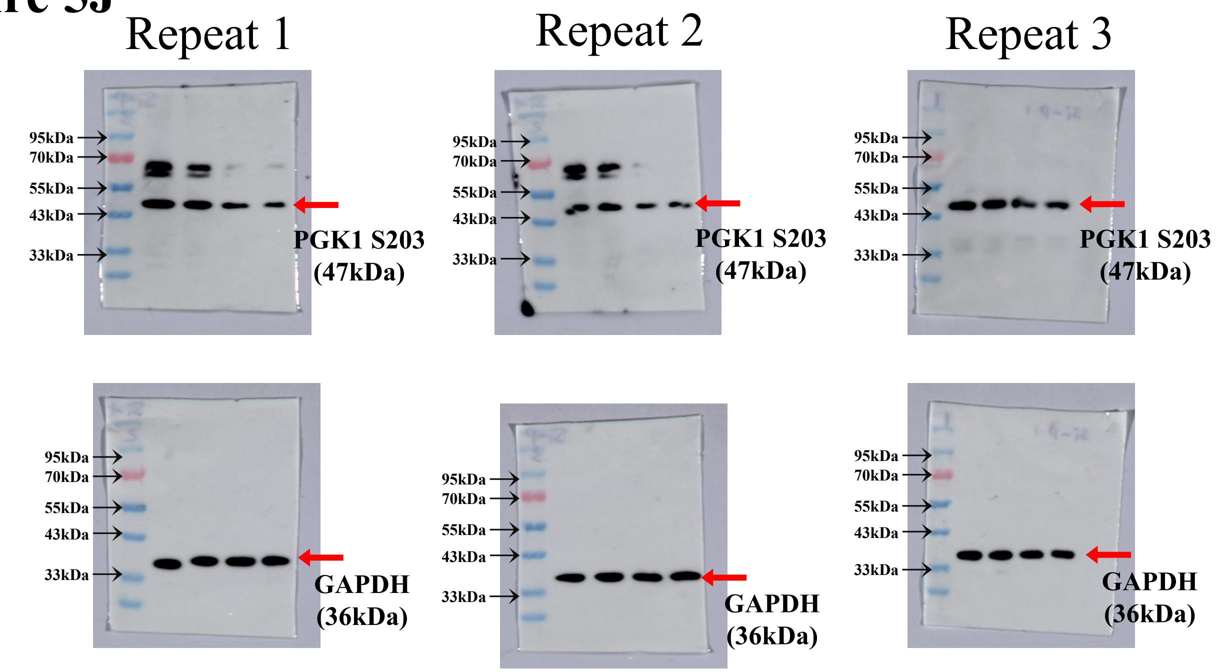

Figure 5K

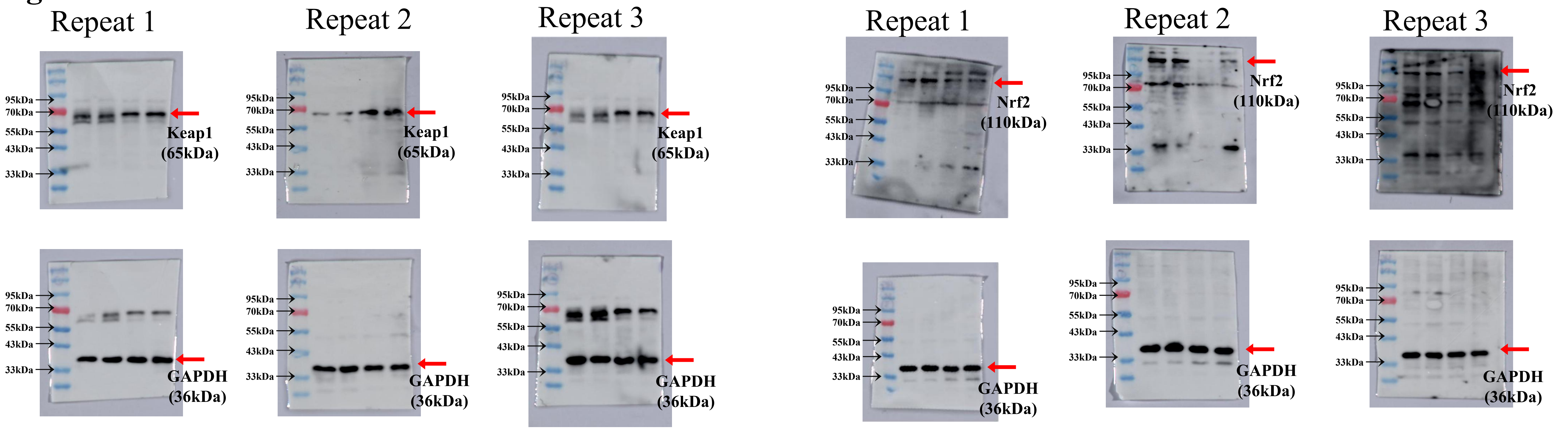

Figure 5L

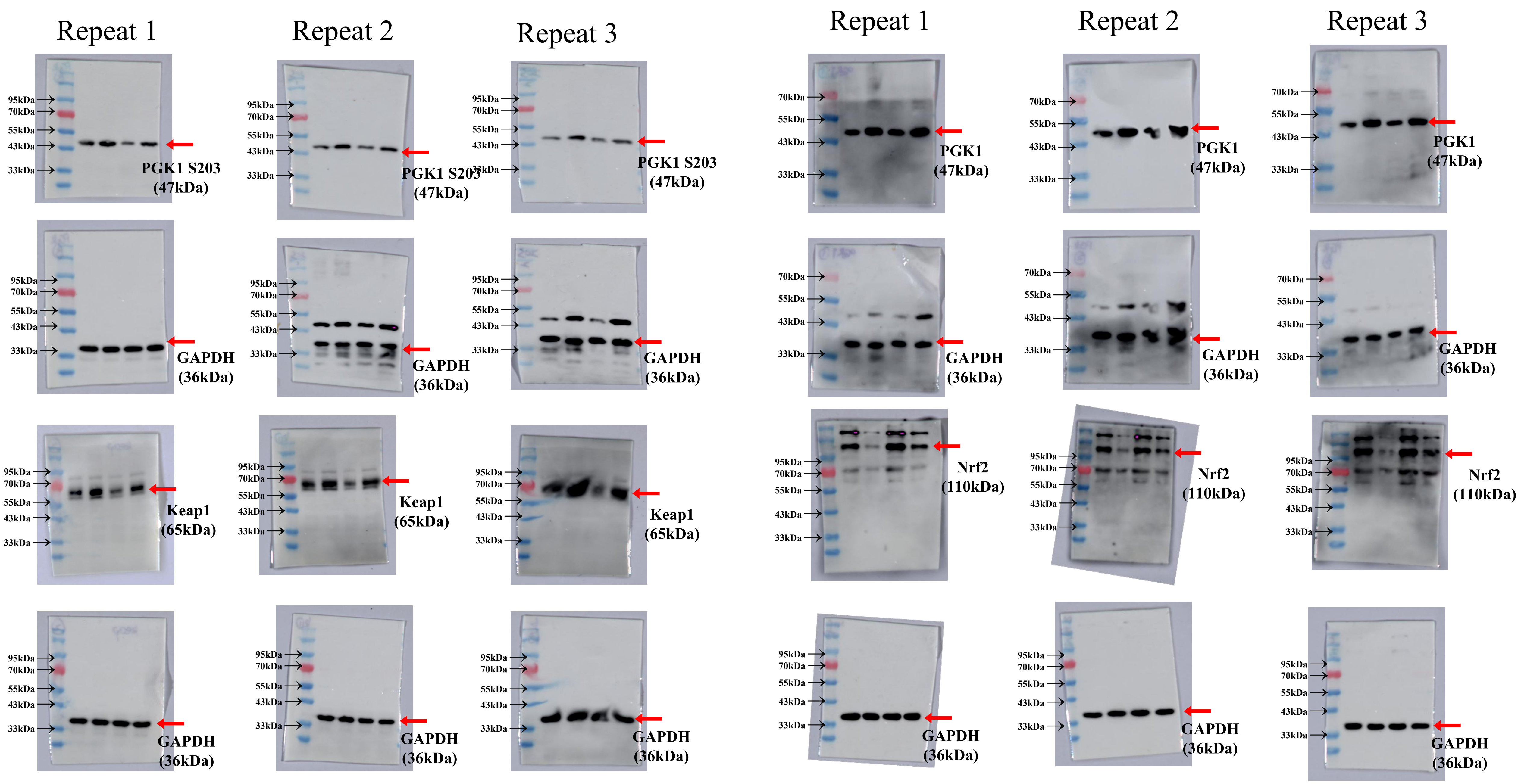

Repeat 1
Repeat 2
Repeat 3
Repeat 1
Repeat 2
Repeat 3
PGK1 S203 (47kDa)
GAPDH (36kDa)
Keap1 (65kDa)
PGK1 (47kDa)
Nrf2 (110kDa)
95kDa
70kDa
55kDa
43kDa
33kDa

Figure 5L

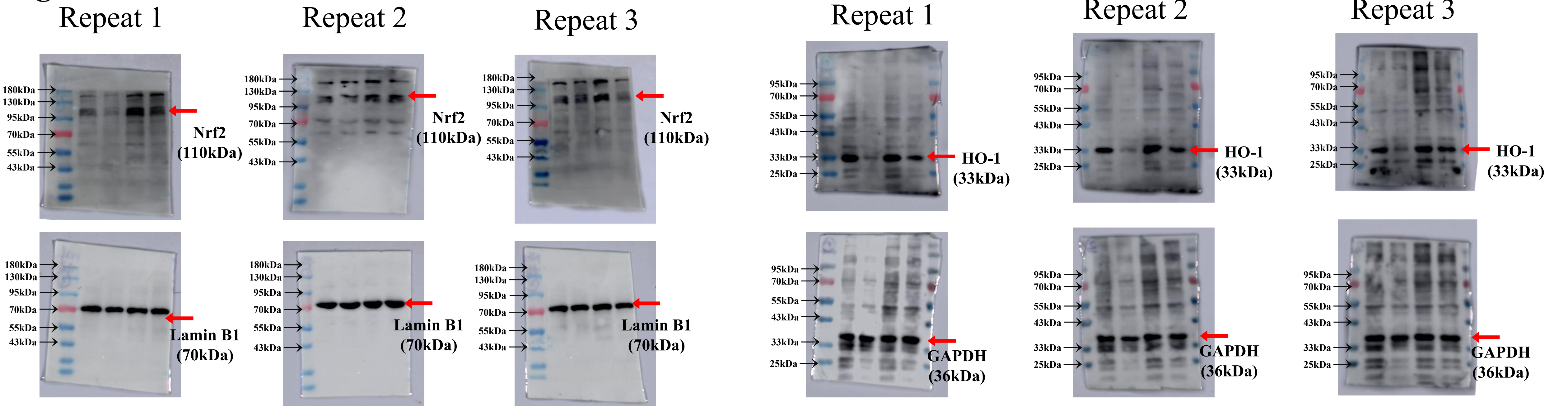

Figure 6A

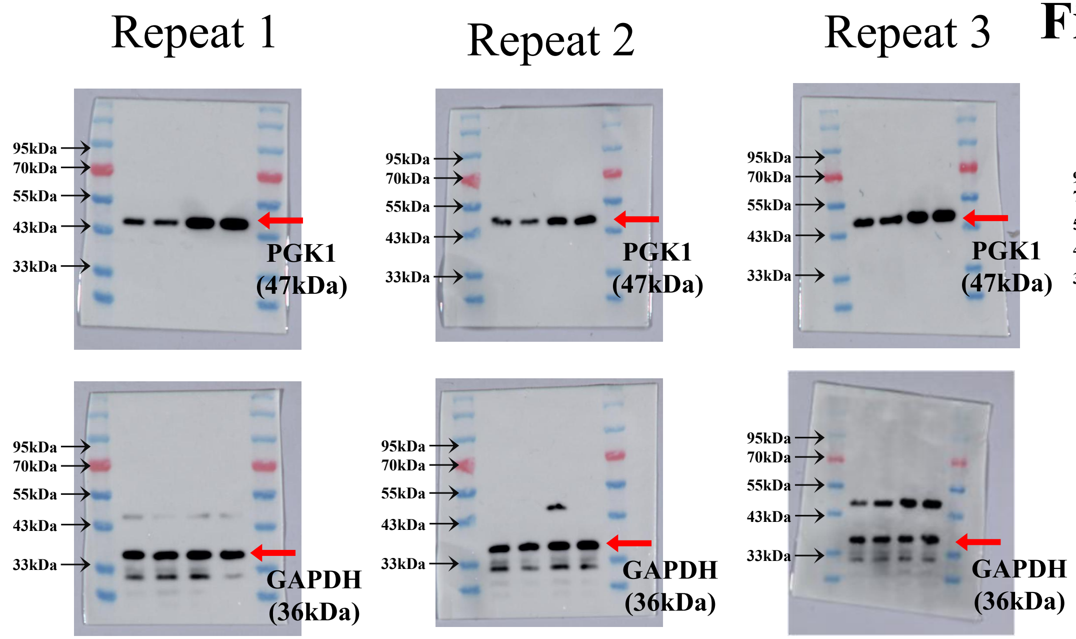

Figure 6C

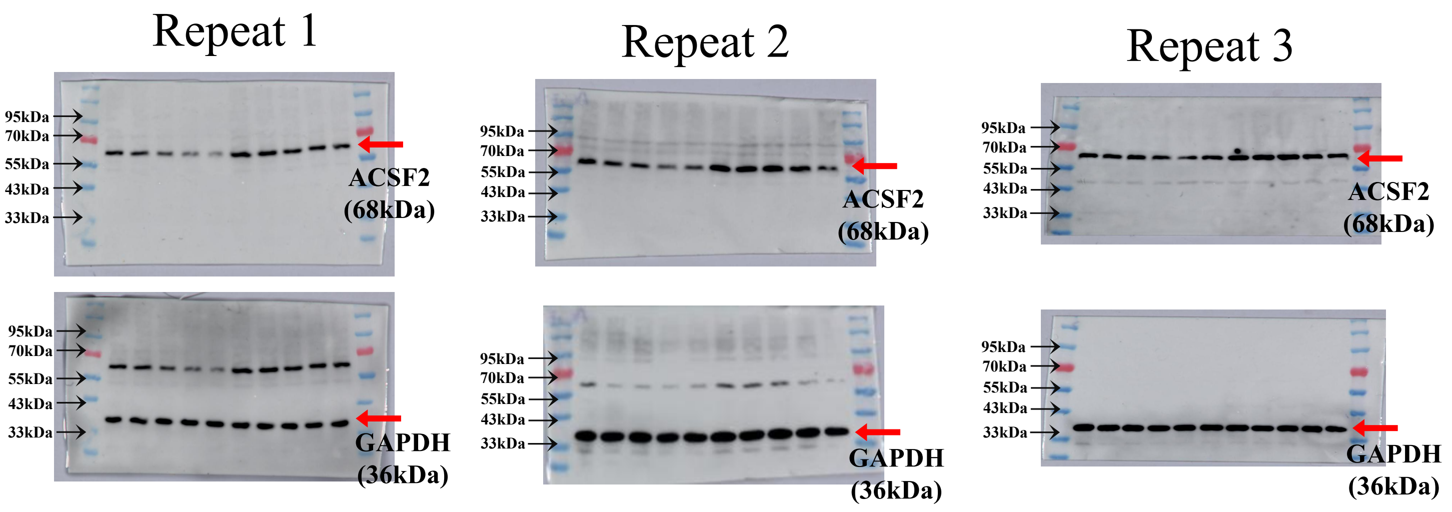

Figure 6D

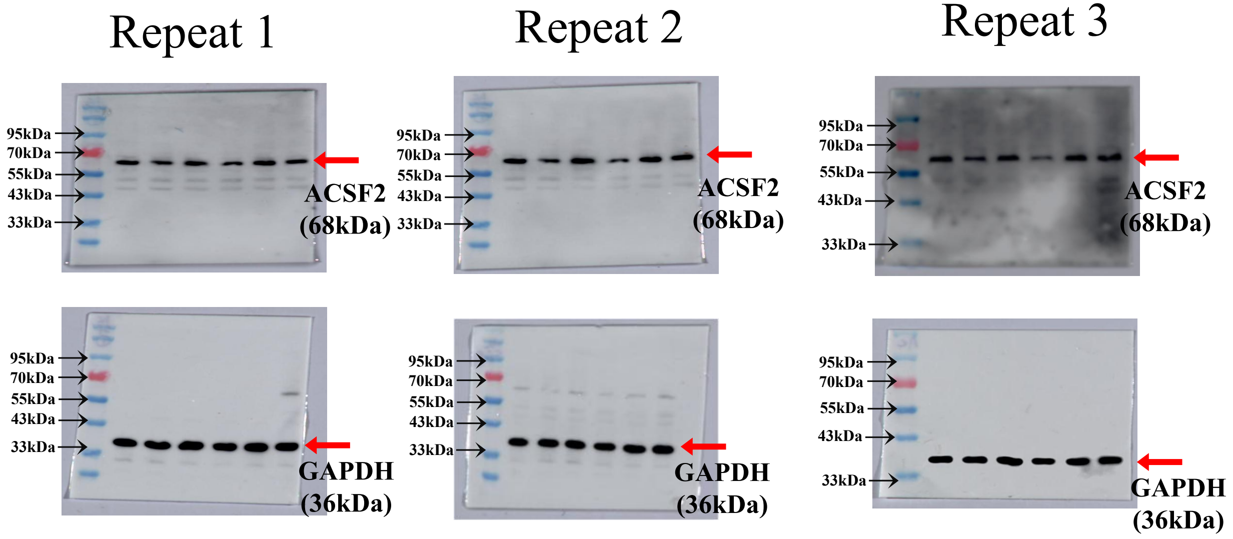

Figure 6E

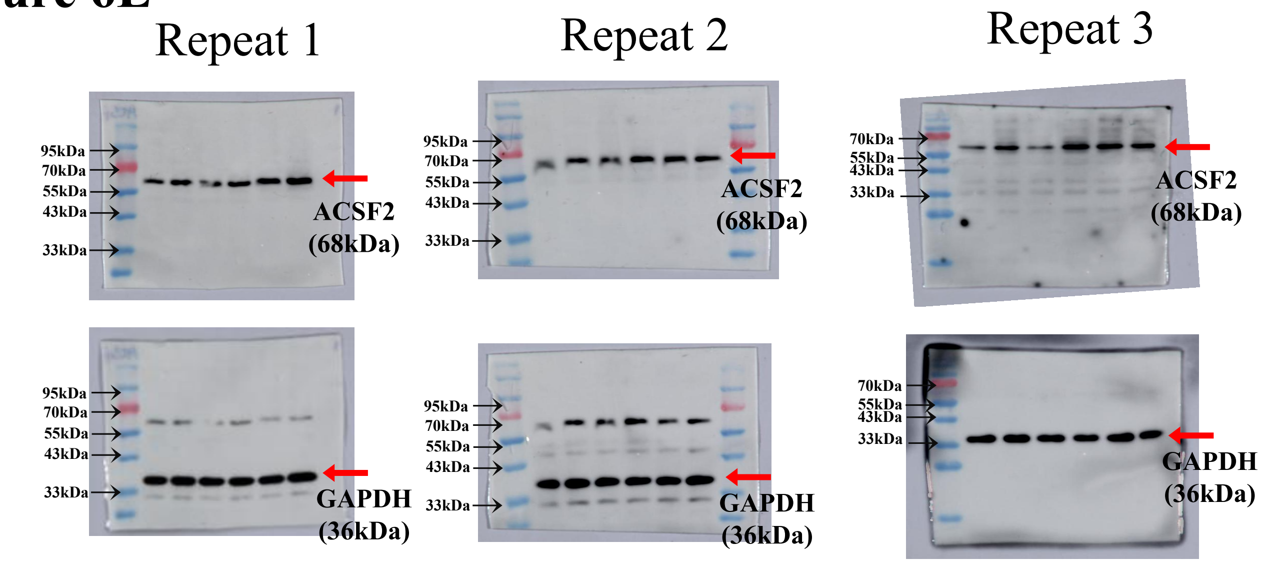

Figure 6F

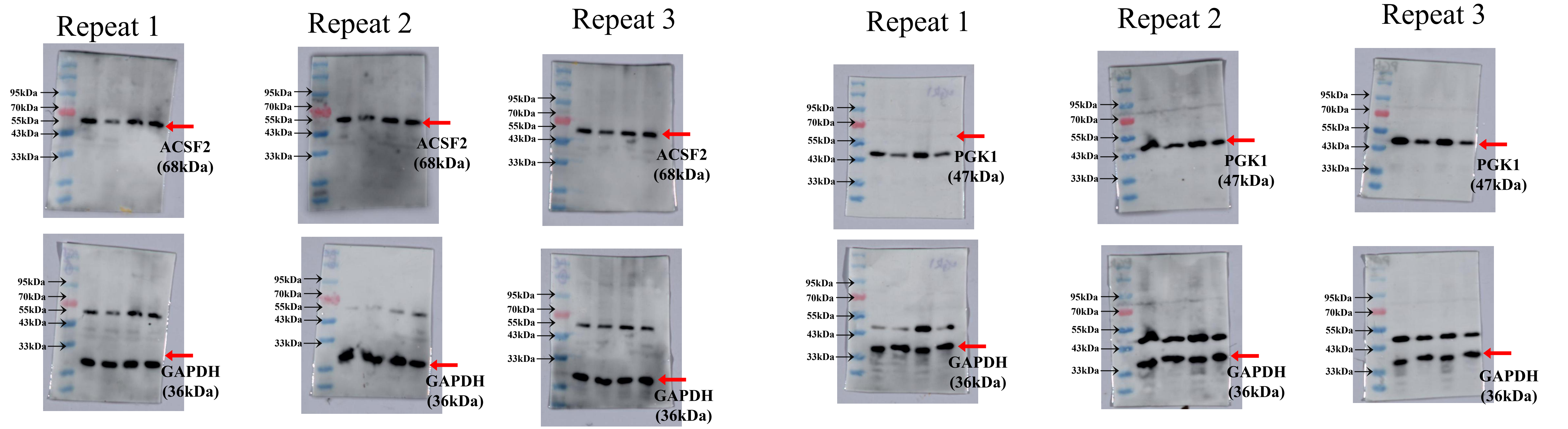

Figure 6G

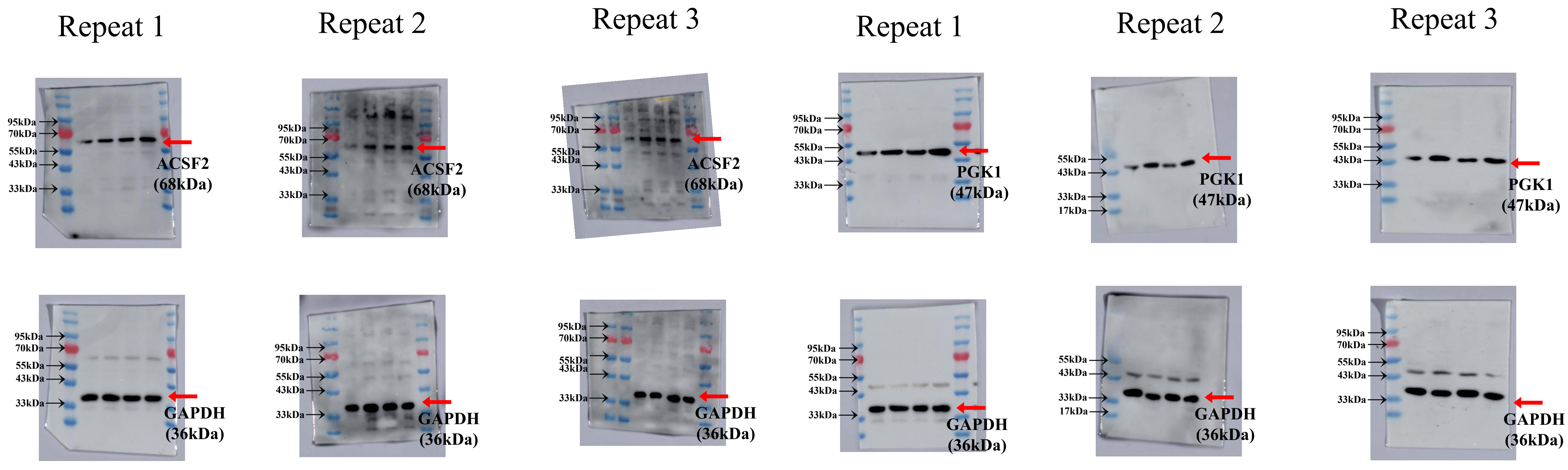
Repeat 1
Repeat 2
Repeat 3
Repeat 1
Repeat 2
Repeat 3
95kDa
70kDa
55kDa
43kDa
33kDa
17kDa
ACSF2
(68kDa)
PGK1
(47kDa)
GAPDH
(36kDa)
