# Supplementary material for: ACSF2-PGK1 interaction promotes ferroptosis in renal tubular epithelial cells of diabetic nephropathy by regulating Keap1/Nrf2 signaling
Source: Redox Rep. 2025 Jul 16;30(1):2529618. doi: 10.1080/13510002.2025.2529618 (PMC12269058; doi:10.1080/13510002.2025.2529618)
Supplement: Supplemental_material_Figure_clean_version.pdf [file YRER_A_2529618_SM9583.pdf]

Fig. S1.

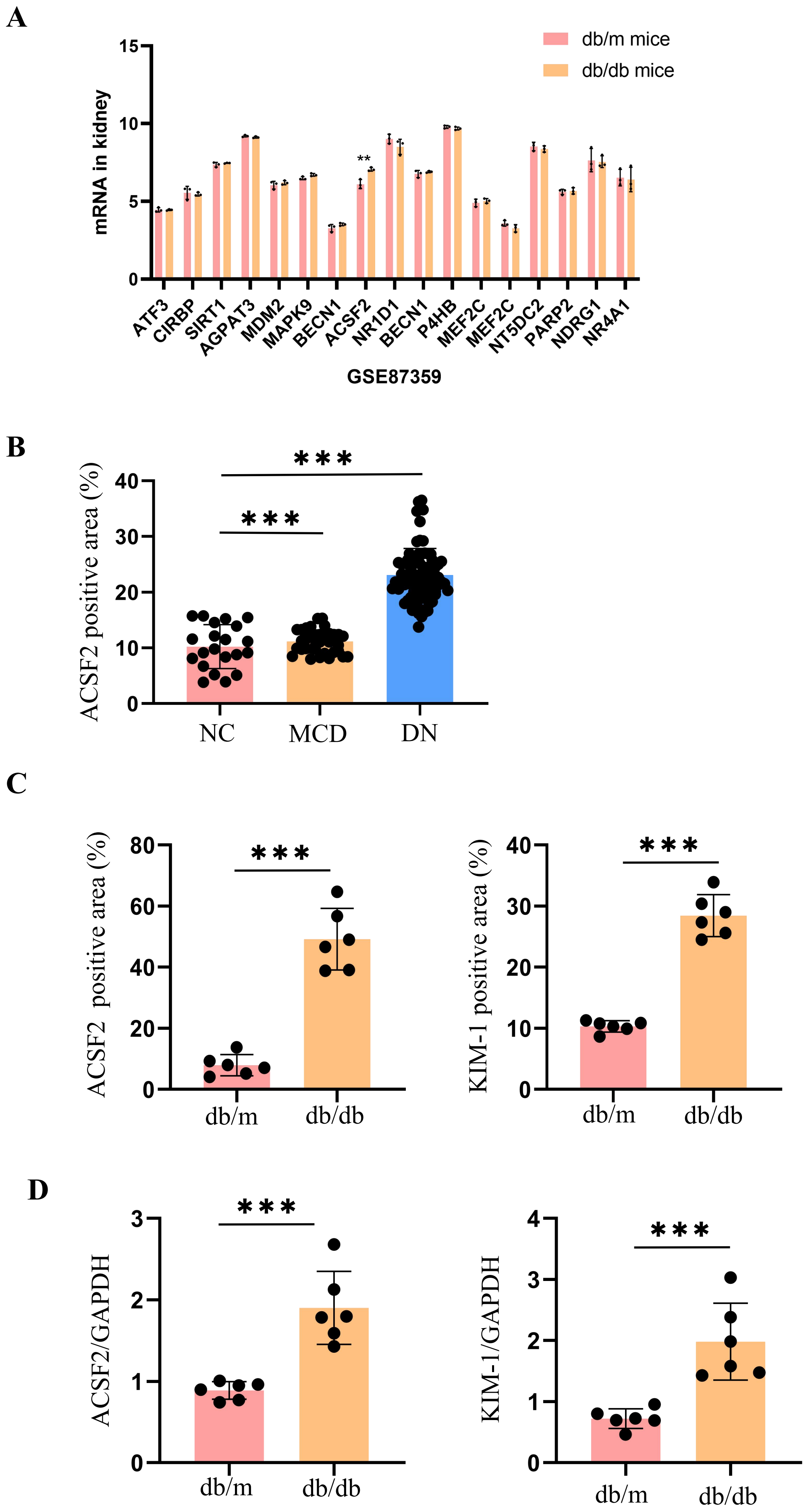

(A). Initial analysis of ferroptosis-related genes expression in kideny of db/db mice was performed using the GSE87359 database. **(B)**. Relative quantification of ACSF2 immunohistochemical staining in DN patients, MCD patients, and normal controls. **(C)**. Relative quantification of ACSF2 and KIM-1 immunohistochemical staining in db/db mice. **(D)**. Quantification of western blot in Figure 1K. The data represent the mean ± SEM. *$p < 0.05$, **$p < 0.01$, ***$p < 0.001$. NC, normal control; DN, diabetic nephropathy; MCD, minimal change disease.

Fig. S2.

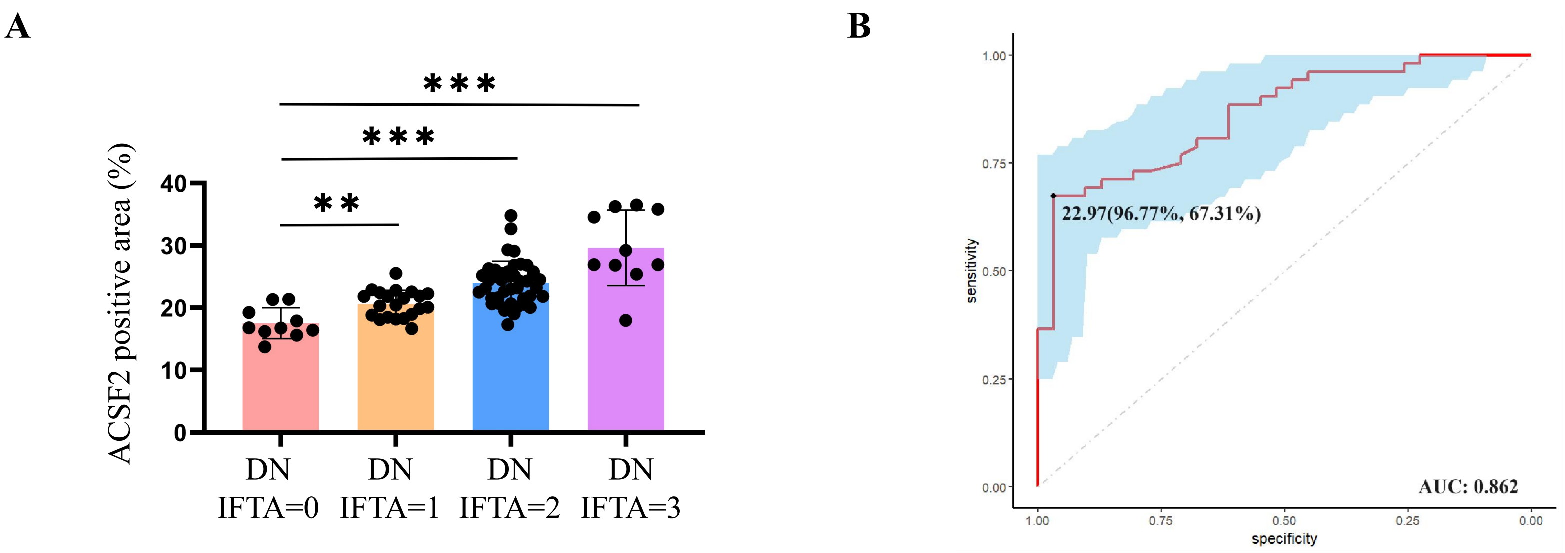

(A). Relative quantification of ACSF2 immunohistochemical staining in DN patients with different IFTA scores. **(B)**. Receiver operating characteristic (ROC) curves for ACSF2. The data represent the mean ± SEM. *p< 0.05, **p< 0.01, ***p< 0.001. DN, diabetic nephropathy. DN, diabetic nephropathy; IFTA, interstitial fibrosis and tubular atrophy.

Fig. S3.

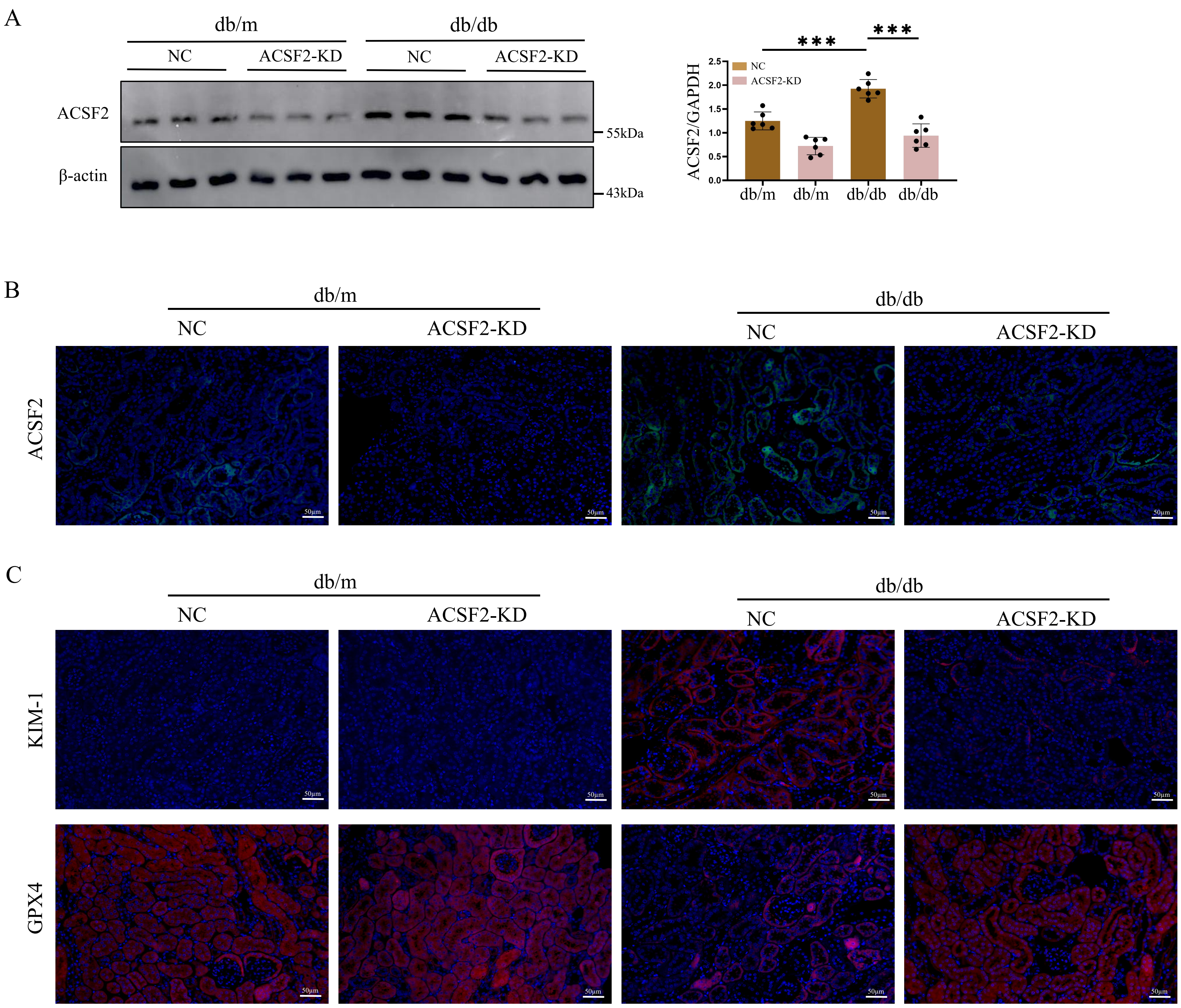

(A). Western blot showing ASCF2 knockdown in the kidney. **(B)**. Representative immunofluorescence staining of ACSF2 in different groups of db/db mice (scale bar=50μm). **(C)**. Representative immunofluorescence staining of KIM-1 and GPX4 in different groups of db/db mice (scale bar=50μm). The data represent the mean ± SEM for 6 mice. *p< 0.05, **p< 0.01, ***p< 0.001.

Fig. S4.

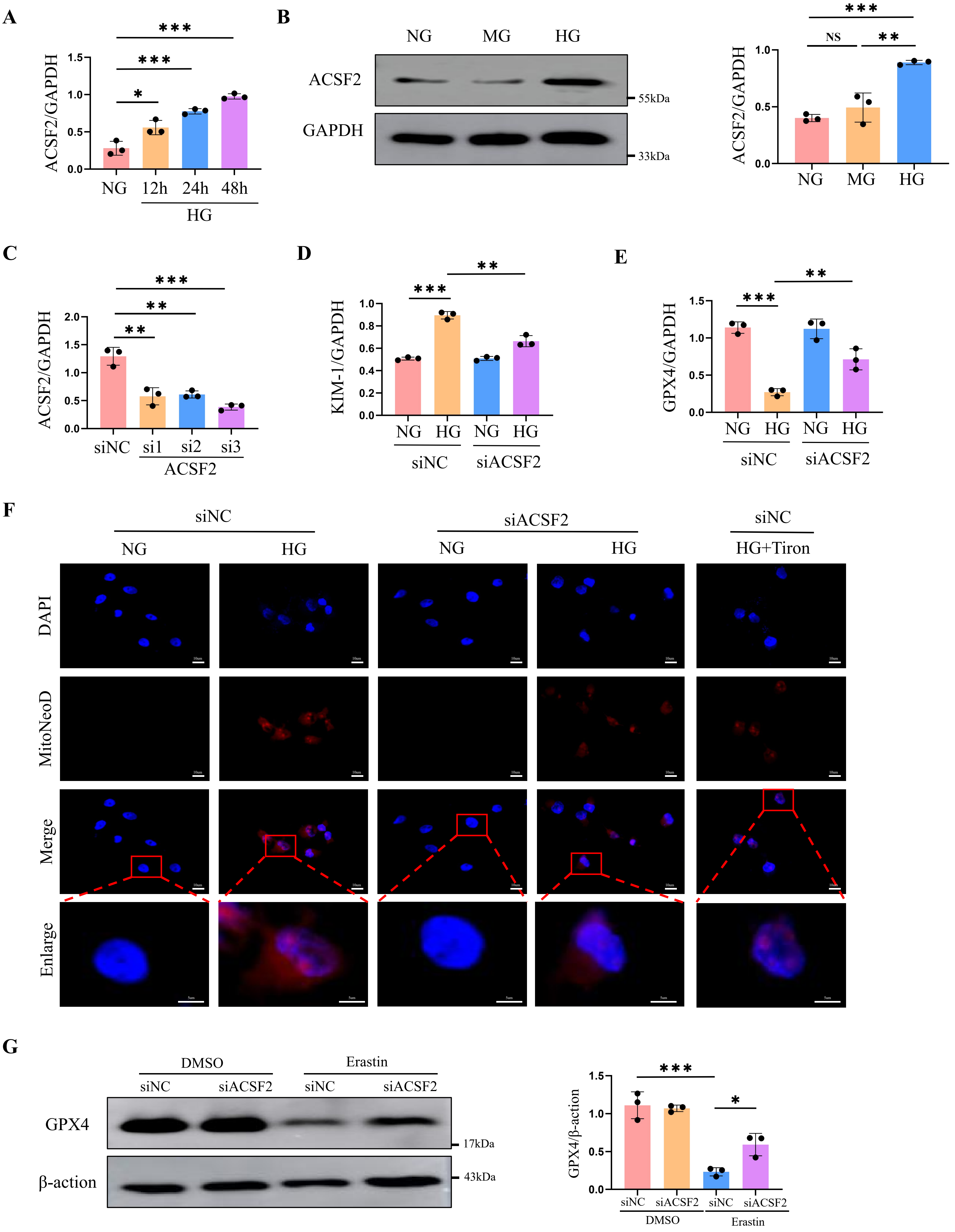

(A). Quantification of western blot in Figure 1A. **(B)**. Western blot analysis and quantification of ACSF2 in HK2 cells after exposure to 30mM HG or 30mM mannitol. **(C, D, E)**. Quantification of western blot in Figure 1C, Figure 1F and Figure 1K. **(F)**. Representative image of MitoNeoD staining in HK-2 cells (scale bar=10μm, Enlarged scale bar=5μm). **(G)**. Western blot analysis and quantification of GPX4 in HK2 cells after exposure to Erastin. The data represent the mean ± SEM. *$p < 0.05$, **$p < 0.01$, ***$p < 0.001$. DN, diabetic nephropathy. HG, high glucose, 30 mmol/L; MG, mannitol, 30 mmol/L; NG, normal glucose, 5.5 mmol/L.

Fig. S5.

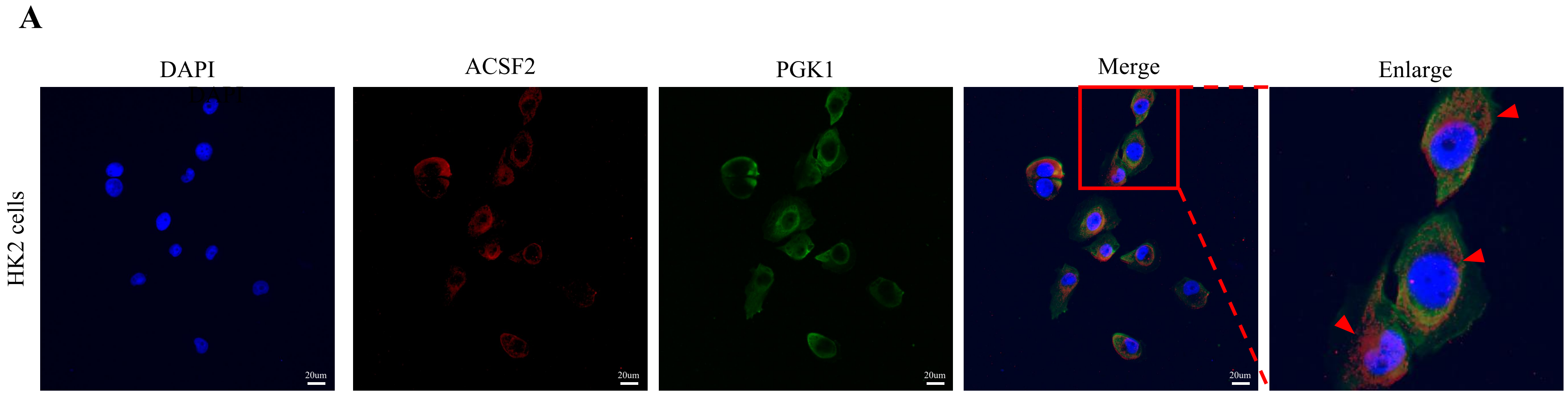

(A). Representative immunofluorescence staining images of ACSF2 (red) and PGK1 (green) in HK2 cells.
