# Supplementary material for: ACSF2-PGK1 interaction promotes ferroptosis in renal tubular epithelial cells of diabetic nephropathy by regulating Keap1/Nrf2 signaling
Source: Redox Rep. 2025 Jul 16;30(1):2529618. doi: 10.1080/13510002.2025.2529618 (PMC12269058; doi:10.1080/13510002.2025.2529618)
Supplement: Supplementary_data_Table_clean_version.pdf [file YRER_A_2529618_SM9582.pdf]

Table S1. General data of NC, MCD and DN patients.

| Characteristic | NC<br>N=21 | MCD<br>N=35 | DN<br>N=83 | P |
|---|---|---|---|---|
| Age (years) | 53(46.5-59) | 49(30-59) | 53(44-58) | 0.202 |
| Gender, male/female | 13/8 | 21/14 | 63/20 | 0.216 |
| BMI(kg/m2) | 25.35(23.44-27.4) | 24.39(22.06-27.73) | 25.35(23.44-27.4) | 0.134 |
| FBG (mmol/l) | 5.38(5.15-5.55) | 4.87(4.52-5.74) | 7.37(5.76-8.99) | <0.001 |
| SBP (mmHg) | 130.9±18.73 | 134.97±19.94 | 144.11±20.24 | 0.008 |
| DBP (mmHg) | 79.14±11.08 | 85.94±11.8 | 85.64±13.09 | 0.087 |
| BUN (mmol/l) | 5.14(4.29-6.55) | 5.44(3.84-7.82) | 7.96(6.06-10.4) | <0.001 |
| Scr (umol/l) | 73(61-85.5) | 73.7(61.2-85.5) | 127.5(87.9-156.7) | <0.001 |
| UA (umol/l) | 322(251-371.5) | 362(268-419) | 385(329-449) | 0.016 |
| eGFR-EPI(ml/min/1.73m$^2$) | 101(92-106) | 96.09(78.94-110.03) | 50.84(39.72-85) | <0.001 |

NC, normal control; DN, diabetic nephropathy; MCD, minimal change disease; BMI: body Mass Index; FBG: fasting blood glucose; SBP: systolic blood pressure; DBP: diastolic blood pressure; BUN: blood urea nitrogen; Scr: serum creatinine; UA: uric acid; eGFR-EPI: using the CKD-EPI equation for estimated glomerular filtration rate (eGFR);
Data are presented as means and standard deviations or medians and interquartile ranges for continuous variables; and as frequencies and percentages for categorical variables; P values were determined with one-way ANOVA tests or Kruskal-Wallis tests for continuous variables and chi-square test for categorical variables

Table S2. Classification of 37 ferroptosis-related DEGs.

| Driver gene | Suppressor gene | Marker gene |
|---|---|---|
| ACSF2 | ABCC5 | FTH1 |
| AGPAT3 | ADAMTS13 | |
| ATF3 | AKT1S1 | |
| ATM | BCAT2 | |
| BECN1 | CDKN1A | |
| CIRBP | GDF15 | |
| EGLN2 | GSTM1 | |
| EGR1 | JUN | |
| FAR1 | MEF2C | |
| GABARAPL1 | NFS1 | |
| KLF2 | NR4A1 | |
| LYRM1 | NT5DC2 | |
| MAPK9 | P4HB | |
| MDM2 | PARP2 | |
| NDRG1 | RELA | |
| NR1D1 | ZFP36 | |
| SAT1 | | |
| SCP2 | | |
| SIRT1 | | |
| WWTR1 | | |

DEG: differentially expressed genes.

Table S3. General data of different type of DN patients.

| Characteristic | IFTA=0<br>N=10 | IFTA=1<br>N=21 | IFTA=2<br>N=42 | IFTA=3<br>N=10 | P |
|---|---|---|---|---|---|
| Age (years) | 46.4±9.43 | 52.33±9.57 | 51.71±9.56 | 56.3±6.67 | 0.129 |
| Sex, male/female | 6/4 | 17/4 | 31/11 | 9/1 | 0.413 |
| BMI (kg/m2) | 26.45(23.72-31.32) | 26.04(23.55-28.24) | 25.7(23.28-27.35) | 24.71(23.32-25.28) | 0.481 |
| Diabetes history (months) | 48(10.5-87) | 72(17-120) | 90(36-147) | 120(72-156) | 0.446 |
| FBG (mmol/l) | 8(5.97-8.89) | 6.35(5.81-7.49) | 8.08(5.73-11.34) | 6.9(4.46-9.35) | 0.208 |
| HbA1c (%) | 6.7(6.05-7.1） | 6.8(6.3-7.45) | 7.3(6.48-8.48) | 7.2(6.58-8.58) | 0.139 |
| SBP (mmHg) | 127±9.74 | 142.57±22.58 | 147.93±19.47 | 148.4±18.95 | 0.945 |
| DBP (mmHg) | 73.5(68.75-80.5) | 86(78.5-95) | 86.5(80.75-96) | 79(73.25-91.75) | 0.008 |
| BUN (mmol/l) | 6.39±2.89 | 7.69±3.22 | 8.85±2.85 | 11.07±4 | 0.006 |
| Scr (umol/l) | 59.6(48.43-79.8) | 89.5(84.75-131.65) | 134.15(117.3-157.78) | 207(179.83-232.38) | <0.001 |
| UA (umol/l) | 365.1±63.87 | 395.62±103.41 | 372.71±110.37 | 437.4±56.65 | 0.262 |
| eGFR-EPI(ml/min/1.73m$^2$) | 110.72(93.87-120.2) | 79.97(48.31-91.85) | 49.05(39.26-61.28) | 28.52(25.97-33.66) | <0.001 |
| Alb (g/l) | 33.55(26.5-41) | 34.7(27.75-38.6) | 34.1(24.03-38.18) | 29.7(19.475-38.35) | 0.609 |
| TC (mmol/l) | 4.705(4.12-5.94) | 5.02(4.07-5.8) | 5.855(4.36-8.42) | 6.075(4.37-8.92) | 0.148 |
| TG (mmol/l) | 1.885(1.63-3.41) | 1.93(1.24-2.76) | 1.76(1.14-2.7) | 1.96(1.1-3) | 0.573 |
| uACR (mg/g) | 244.2（139.35-527.94） | 281.9（169.46-1606.35） | 1476.58（193.97-4043.35） | 2578.65（857.51-4012.45) | <0.001 |
| uRBP (mg/l) | 0.24(0.05-0.49) | 0.37(0.22-2.2) | 3.57(0.96-8.42) | 7.58(5.52-10.03) | <0.001 |
| uNAG (U/l) | 8.55(7.7-11.36) | 13.67(9.65-15.06) | 14.9(12.24-21.3) | 22.66(16.39-24.92) | <0.001 |
| β2-MG (mg/l) | 0.29(0.16-0.34) | 0.39(0.1-1.43) | 1.21(0.41-2.97) | 9(1.34-30.35) | 0.001 |

| | | | | | |
|---|---|---|---|---|---|
| α1-MG (mg/l) | 11.04(8.64-11.83) | 22.9(10.43-29.83) | 26.9(20.43-43.5) | 55.47(25.26-68.65) | <0.001 |
| Pathological type, I/II/III/IV | 5/5/0/0 | 1/14/6/0 | 0/24/14/4 | 0/0/3/7 | <0.001 |
| Inflammation score, grade 0/1/2 | 6/4/0 | 0/21/0 | 0/16/26 | 0/10/0 | <0.001 |
| Arteriole hyalinosis score, grade 0/1/2 | 5/5/0 | 0/16/5 | 0/16/26 | 0/0/10 | <0.001 |
| Atherosclerosis score, grade 0/1/2 | 5/5/0 | 0/19/2 | 0/23/19 | 0/0/3/7 | <0.001 |

IFTA, interstitial fibrosis and tubular atrophy; DN, diabetic nephropathy; BMI: body Mass Index; FBG: fasting blood glucose; HbA1c: glycosylated hemoglobin; SBP: systolic blood pressure; DBP: diastolic blood pressure; BUN: blood urea nitrogen; Scr: serum creatinine; UA: uric acid; eGFR-EPI: using the CKD-EPI equation for estimated glomerular filtration rate (eGFR); Alb: serum albumin; TC: total blood cholesterol; TG: blood triglycerides; uTCR: urine total protein-to-creatinine ratio; uACR: urine albumin-to-creatinine ratio; uRBP: urine retinol-binding protein, uNAG: urine N-acetyl-β-d-glucosaminidase; u-β2 -MG: urine β2-microglobulin; u-α1 -MG: urine α1-microglobulin.

Data are presented as means and standard deviations or medians and interquartile ranges for continuous variables; and as frequencies and percentages for categorical variables; P values were determined with one-way ANOVA tests or Kruskal-Wallis tests for continuous variables and chi-square test for categorical variables
